# Impaired spike-gamma coupling of area CA3 fast-spiking interneurons as the earliest functional impairment in the *App*^*NL-G-F*^ mouse model of Alzheimer’s disease

**DOI:** 10.1038/s41380-021-01257-0

**Published:** 2021-08-12

**Authors:** Luis Enrique Arroyo-García, Arturo G. Isla, Yuniesky Andrade-Talavera, Hugo Balleza-Tapia, Raúl Loera-Valencia, Laura Alvarez-Jimenez, Giusy Pizzirusso, Simone Tambaro, Per Nilsson, André Fisahn

**Affiliations:** 1 Neuronal Oscillations Laboratory; Division of Neurogeriatrics; Center for Alzheimer Research; Department of Neurobiology, Care Sciences and Society, Karolinska Institutet, Solna, Sweden; 2 Division of Neurogeriatrics; Center for Alzheimer Research; Department of Neurobiology, Care Sciences and Society, Karolinska Institutet, Solna, Sweden

**Keywords:** Neuroscience, Diseases

## Abstract

In Alzheimer’s disease (AD) the accumulation of amyloid-β (Aβ) correlates with degradation of cognition-relevant gamma oscillations. The gamma rhythm relies on proper neuronal spike-gamma coupling, specifically of fast-spiking interneurons (FSN). Here we tested the hypothesis that decrease in gamma power and FSN synchrony precede amyloid plaque deposition and cognitive impairment in *App*^NL-G-F^ knock-in mice (*App*^NL-G-F^). The aim of the study was to evaluate the amyloidogenic pathology progression in the novel *App*^NL-G-F^ mouse model using in vitro electrophysiological network analysis. Using patch clamp of FSNs and pyramidal cells (PCs) with simultaneous gamma oscillation recordings, we compared the activity of the hippocampal network of wild-type mice (WT) and the *App*^NL-G-F^ mice at four disease stages (1, 2, 4, and 6 months of age). We found a severe degradation of gamma oscillation power that is independent of, and precedes Aβ plaque formation, and the cognitive impairment reported previously in this animal model. The degradation correlates with increased Aβ_1-42_ concentration in the brain. Analysis on the cellular level showed an impaired spike-gamma coupling of FSN from 2 months of age that correlates with the degradation of gamma oscillations. From 6 months of age PC firing becomes desynchronized also, correlating with reports in the literature of robust Aβ plaque pathology and cognitive impairment in the *App*^NL-G-F^ mice. This study provides evidence that impaired FSN spike-gamma coupling is one of the earliest functional impairment caused by the amyloidogenic pathology progression likely is the main cause for the degradation of gamma oscillations and consequent cognitive impairment. Our data suggests that therapeutic approaches should be aimed at restoring normal FSN spike-gamma coupling and not just removal of Aβ.

## Introduction

Alzheimer’s disease (AD) is the main cause of dementia and a major health concern for society. Despite huge efforts the exact cause of sporadic AD remains unknown and lack of tools for early detection complicates even symptomatic treatment [1]. On the molecular level AD involves increased levels, misfolding and aggregation of amyloid-β peptide (Aβ), mainly Aβ_1-40_ and Aβ_1-42_ with an increased Aβ_1-42/_Aβ_1-40_ ratio [1–3]. Studies have revealed that excessive amounts of Aβ in the brain cause neuroinflammation, decrease glutamatergic synaptic transmission, cause neuronal and synaptic loss, changes in synaptic plasticity and disruption of brain oscillations [3–6]. Moreover, clinical data have shown a correlation between cognitive deterioration and the disruption of cognition-relevant brain oscillations in the gamma frequency-band (30–80 Hz) [7–10].

Gamma oscillations are well described in different brain regions and are known to act as a temporal scaffold for neuronal communication and information processing [10]. In brief, gamma oscillations are the result of the rhythmic synchronization of action potentials generated by excitatory pyramidal cells (PC) and inhibitory fast-spiking interneurons (FSN), and the resultant rhythmic postsynaptic currents. These currents can be recorded macroscopically in electroencephalograms, or invasively as local field potentials (LFP) [7, 10]. In the healthy human brain, neocortical and hippocampal gamma oscillations are important for cognitive processes such as the formation of new memories [11–13]. In particular, area CA3 of the hippocampus has been described as a gamma oscillation generator, in part due to the local recurrent excitatory connections between PCs, which allow for the generation of greater power oscillations in comparison with other regions such as CA1 [14]. In addition, the close communication with the dentate gyrus establishes a local circuitry involved in episodic memory [15–17].

Recent evidence establishes that inhibitory FSN regulate oscillatory rhythms and network synchrony, suggesting that FSN are a main component in the generation and maintenance of the gamma rhythms and hence are important for cognitive performance [10, 18]. In contrast, the pathological misfolding and aggregation of Aβ has been linked to the degradation of gamma oscillations in AD patients [8, 9], which occurs long before the first signs of cognitive impairment [19, 20]. Using an acute ex vivo AD mouse model we have previously demonstrated that Aβ in pathophysiological concentrations degrades mouse hippocampal gamma oscillation within tens of minutes, causing desynchronization and an excitatory/inhibitory imbalance in PC [4, 21]. Studies using different transgenic mice that overproduce the amyloid-β precursor protein (APP) found functional impairments during the progression of Aβ pathology [22–26]. Collectively, these studies have shown that Aβ affects inhibitory interneurons, specifically interneurons expressing Nav1.1 receptors (voltage-gated sodium channel), which are responsible for the regulation of oscillatory rhythms [18, 22, 27]. In fact, treatments manipulating inhibitory interneurons have managed to restore gamma oscillations and showed significant improvement in cognitive behaviors of APP transgenic mice [18, 24, 28, 29].

Although APP-overexpressing transgenic mice have been important tools in AD research, concerns exist regarding the interference of the unphysiologically high levels of APP and its proteolytic fragments (which are not increased in AD in humans) with normal brain function, and the creation of artificial phenotypes [30, 31]. Here, we overcome this problem using a novel *App* knock-in mouse model that utilizes the endogenous mouse APP gene carrying the Swedish KM670/671NL (NL), the Artic E693G (G) and the Beyreuther/Iberian I716F (F) mutations with a humanized Aβ sequence (*App*^NL-G-F^) [30, 31]. This novel mouse model starts to show amyloid plaque formation at 3 months of age and behavioral impairment and neuroinflammation at 6 months of age [30–32]. We used this model to test the hypothesis that decrease in gamma oscillation power and FSN synchrony precede amyloid plaque deposition and cognitive impairment in *App*^NL-G-F^ mice.

Our results show that degradation of gamma oscillations is independent of, and precedes, Aβ plaque formation but correlates with the increase of Aβ_1-42_ concentration. Impaired FSN spike-gamma coupling occurs as early as 2 months of age when signs of neuroinflammation are still absent. From 6 months of age PC spike-gamma coupling becomes impaired also, matching the full establishment of Aβ plaques, evident gliosis, and cognitive impairment. This study provides evidence that impaired FSN spike-gamma coupling is the earliest functional impairment in AD progression leading to degradation of gamma oscillations and cognitive impairment, and suggests that therapeutic approaches in AD should be aimed at restoring normal FSN spike-gamma coupling and not just removal of Aβ.

## Methods

### Animals

Experiments were performed in accordance with the ethical permit granted by Norra Stockholm’s Djurförsöksetiska Nämnd (dnr N45/13) to AF. Thirty-seven wild-type (WT) C57/BL6 male mice were supplied by Charles River, Germany. Thirty-four homozygous *App*^NL-G-F^ mice [30] were obtained from local breeding using the C57/BL6 strain as background (ethical approval ID 407 from Linköping’s animal ethical board). Four age points were randomly selected for our experiments: 1 month old (m.o.; 29–32 postnatal day; WT *n* = 5, *App*^NL-G-F^ *n* = 4), 2 m.o. (70–80 postnatal day; WT *n* = 11, *App*^NL-G-F^ *n* = 11), 4 m.o. (115–125 postnatal day; WT *n* = 11, *App*^NL-G-F^ *n* = 10) and 6 m.o. (175–185 postnatal day; WT *n* = 10, *App*^NL-G-F^ *n* = 9). For brain dissection mice were deeply anaesthetized with isoflurane before being sacrificed by decapitation. Experiments were performed by an investigator blinded to the group allocation.

### Drugs and chemicals

Chemical compounds used in intracellular and extracellular solutions were obtained from Sigma-Aldrich Sweden AB (Stockholm, Sweden). Kainic acid (KA), 6,7-Dinitroquinoxaline-2,3(1H,4H)-dione (DNQX), D-(-)-2-Amino-5-phosphonopentanoic acid (D-APV), bicuculline and Tetrodotoxin (TTX) were obtained from Tocris Bioscience (Bristol, UK).

### Hippocampal slice preparation

After decapitation the brain was quickly dissected out and placed in ice-cold artificial cerebrospinal fluid (ACSF) prepared for dissection and containing (in mM): 80 NaCl, 24 NaHCO_3_, 25 glucose, 1.25 NaH_2_PO_4_, 1 ascorbic acid, 3 Na-pyruvate, 2.5 KCl, 4 MgCl_2_, 0.5 CaCl_2_, 75 sucrose and bubbled with carbogen (95% O_2_ and 5% CO_2_). Horizontal hippocampal sections 350 µm thick were obtained from both hemispheres with a Leica VT1200S vibratome (Leica Microsystems). Slices were transferred for recovery to an interface holding chamber containing standard recording ACSF (in mM): 124 NaCl, 30 NaHCO_3_, 10 glucose, 1.25 NaH_2_PO_4_, 3.5 KCl, 1.5 MgCl_2_, 1.5 CaCl_2_, and kept for at least 1 h prior to any recordings. During recovery, temperature was held at 37 °C for the first 15 min and then allowed to cool down to room temperature. Slices in the holding chamber were continuously supplied with humidified carbogen gas (5% CO_2_, 95% O_2_). Local field potentials (LFP) and neuronal dynamics studies were performed in submerged-type recording chambers, where slices were continuously superfused with oxygenated ACSF at a perfusion rate of 3 ml/min at 34 °C.

### Electrophysiological recordings

LFP and single-cell patch clamp recordings were carried out in hippocampal area CA3. LFP recordings were performed in stratum pyramidale with borosilicate glass microelectrodes, pulled to a resistance of 3–6 MΩ and filled with standard ACSF. Action potentials, I–V and excitatory postsynaptic potentials (EPSCs) were recorded in whole-cell mode with an internal recording solution containing (in mM): 122.5 K^+^-gluconate, 8 KCl, 2 Mg^2+^ ATP, 0.3 Na^+^ GTP, 10 HEPES, 0.2 EGTA, 2 MgCl. For inhibitory postsynaptic potential (IPSC) recordings the internal solution contained (in mM) 140 CsMetSO_4_, 2 Mg^2+^ ATP, 0.3 Na^+^ GTP, 10 HEPES, and 0.6 EGTA. Both solutions were adjusted to pH 7.2–7.3 and osmolarity to 270–280 mosmol/l. I–V, EPSCs and IPSCs were recorded in voltage-clamp configuration with the holding membrane potential (Em) at −70 mV for I–V and EPSCs, and at 0 mV for IPSCs. Action potentials and membrane potential were recorded in gap-free current-clamp mode. Cells were visualized under an upright microscope using IR-DIC microscopy (Axioskop, Carl Zeis AG, Göttingen, Germany).

FSN (Fig. 2a) and PC (Fig. 3a) were identified based on their location, their morphology and their unique electrophysiological characteristics [33, 34]. To confirm the type of neuron we performed three different voltage-response protocols. Firstly, we applied a two-voltage step protocol: one step of 300 pA ms and the other of −150 pA of 400 ms-length each. Secondly, we applied a burst-eliciting protocol: one step of 100 pA over 200 ms followed by a summative step of 300 pA over 50 ms, and then stepping-back to 100 pA for another 200 ms. Thirdly, we applied a ramp protocol starting a voltage ramp of 500 pA at −150 pA for a duration of 400 ms. FSN were mainly found in the stratum radiatum, in close proximity to stratum pyramidale of the CA3 region of the hippocampus (Fig. 2a right). They were only accepted as FSN when their firing characteristics were similar to Fig. 2a (left side). PC were found in the stratum pyramidale of the CA3 region of the hippocampus (Fig. 3a right) and they were only considered for analysis when their firing characteristics were similar to Fig. 3a (left side).

Recordings of I–V relationships were performed by adding blockers to the bath (in µM): 10 bicuculline, 50 APV, 20 DNQX and 1 TTX. LFP gamma oscillations were induced at 34 °C by bath applying 100 nM KA [4] and were allowed to stabilize for at least 30 min. LFP and whole-cell recordings were performed with a patch clamp amplifier (Multiclamp 700B), and data were acquired using pCLAMP 10.4 software (Molecular Devices). LFPs were conditioned using a HumBug 50 Hz noise eliminator (Quest Scientific). All signals were low-pass filtered at 1 kHz, acquired at 5 kHz, digitized and stored using Digidata 1322 A and pCLAMP 10.4 software (Molecular Devices, CA, USA).

### Data analysis

For oscillation power spectra Fast Fourier Transformations were obtained from 60 s of LFP recording (segment length 8192 points) using Axograph X software (Kagi, Berkeley, CA, USA). Frequency variance data was obtained from the power spectra described above using Axograph X. Gamma power was calculated by integrating the power spectral density from 20 to 80 Hz using Clampfit 10.4. Recordings were normalized to the aged-matched WT to minimize variation.

EPSCs and IPSCs were detected off-line using a custom-made template in Clampfit 10.4 software including no <20 averaged representative events. EPSCs and IPSCs parameters such as event amplitude and frequency were analyzed using GraphPad Prism (GraphPad Software, USA). I–V relationships were evaluated from the response to 15 voltage steps (from −90 to 50 mV) and analyzed using Clampfit 10.4.

Spike phase-coupling analysis was performed on concomitant LFP recordings and single-cell recordings using custom-made routines in MATLAB to relate PC or FSN spiking activity to ongoing gamma oscillations [33]. LFP traces were previously filtered using a Butterworth band-pass filter in both directions set to 20–40 Hz (low and high-pass) as the oscillation power spectral analysis indicated that the bulk of the signal lies within this range. AP were detected using an amplitude threshold. The instantaneous phase-angle of gamma oscillations at which AP occurred was determined using a Hilbert transform. Phase-angles of all APs fired and gamma oscillation-phases are represented in polar-plots and expressed in radians with the peak of the oscillation cycle corresponding to 0 π and the trough to ±π. Each action potential was assigned a vector of length 1 with an angle corresponding to the phase of the field at the same time. Once all the vectors were assigned, an averaged resultant phase-density vector was calculated to describe the preferred phase-of-firing (phase-angle) and how recurrent the firing at that angle is (vector length). Longer vector length denotes more synchronized AP firing. Vector length is shown normalized by the total number of APs for each condition for each cell. The preferred phase-angle was determined cell by cell and calculated by averaging the AP phase-angles at which each cell fired for each condition.

All concomitant recordings were tested for circular uniformity using Rayleigh’s test in order to determine whether neurons fired in a phase-related manner. Only recordings with Rayleigh’s *p* values below 0.05 were considered for the analysis. It is worth to mention that after the challenging and highly technical electrophysiological methodology to patch neurons in mature brain and after the selection of neurons and Rayleigh’s test, around 30% of the total could be used for the statistical analysis.

### Immunoblotting analysis

Western blot analysis was carried out as previously reported [35]. Thick sections of cortex-hippocampus were snap-frozen in liquid nitrogen. Solubilization of total protein was carried out using a detergent-based lysis buffer containing 40 mM Tris-HCl (pH 6.8), 1% NP-40, a protease inhibitor cocktail (Sigma-Aldrich) and phosphatase inhibitors (20 mM b-glycerophosphate, 2 nM okadaic acid, 50 mM NaF, 1 mM Na3VO4 from ROCHE). Protein was separated using SDS-PAGE and transferred to a nitrocellulose membrane (Schleicher & Schuell, Germany). Following the transfer, skimmed milk-blocked blots were incubated with the following antibodies: mouse anti-glial fibrillary acidic (GFAP; BD biosciences), rabbit anti- Ionized calcium biding adaptor (IBA1; Novus) and mouse anti- glyceraldehyde-3-phosphate dehydrogenase (GAPDH; Sigma) overnight. Secondary incubation was done using IR-Dye-coupled anti-rabbit or anti-mouse IgG at a 1:15000 dilution at room temperature in darkness (LI-COR). Immunoreactivity was detected by the Odyssey-IR detection system (LI-COR). Some immunoblots were stripped using Restore^™^ Western Blot Stripping buffer (Pierce) at room temperature for 15 min and then blocked and re-blotted with other antibodies. We used ImageJ to perform densitometric analysis on our blots, which were normalized against GAPDH signal to control protein loading in the membranes.

### Statistical analysis

All statistical analyses were performed using GraphPad Prism 8.0. We performed a statistical post-hoc power analysis using G*Power 3 calculator [36]. We performed two-sided statistical analyses comparing the *App*^NL-G-F^ group vs the WT at each age. We used a normality and lognormality test (Shapiro–Wilk test) and we excluded outliers after ROUT (*Q* = 1%) analysis. We performed two-way ANOVA followed by a Holm–Sidak’s multiple comparisons test. Data from the graphs is presented in Supplementary Table 1. In the graphs data are reported as means ± SEM, n’s are specified in each graph and significance levels are *p < 0.05, **p < 0.01, ***p < 0.001, **** *p* < 0.0001.

## Results

### Early degradation of gamma oscillations in App^NL-G-F^ mice

Previously, we have shown that acute short-term (15 min) exposure of the hippocampal network in brain slices of WT mice to Aβ at pathophysiological concentrations (50 nM) dramatically reduces gamma oscillation power, and that this is caused by the impaired spike-gamma coupling of PC and FSN AP generation [4, 21]. Using the *App*^NL-G-F^ (*App* knock-in mouse model) we aimed to test how early in the progression of Aβ pathology we could detect the same effect and the underlying cellular mechanism of functional impairment. For this purpose, we used acute hippocampal slices of *App*^NL-G-F^ mice and characterized neuronal network gamma oscillations as well as PC and FSN behavior before (1 and 2 months of age) and after the onset of Aβ plaque pathology (4 and 6 months of age) [30–32].

**Fig. 1:**
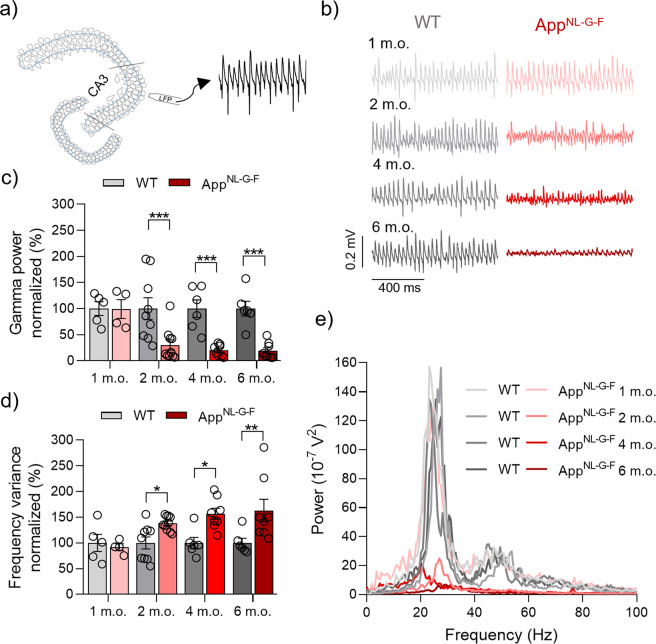
Early degradation of KA-induced gamma oscillations in *App*^NL-G-F^ mice. **a** Representative diagram of LFP electrode location in CA3 region of hippocampal acute slices. **b** Representative sample traces of LFP recordings from WT (left side) and *App*^NL-G-F^ (right side) at 1 m.o., 2 m.o., 4 m.o. and 6 m.o. **c** Summary of normalized gamma oscillation power at 1 m.o. (WT *N* = 5 vs *App*^NL-G-F^ *N* = 4, *p* = 0.9724), 2 m.o. (WT *N* = 9 vs *App*^NL-G-F^ *N* = 10, *p* = 0.0006), 4 m.o. (WT *N* = 6 vs *App*^NL-G-F^ *N* = 8, *p* = 0.0006) and 6 m.o. (WT *N* = 6 vs *App*^NL-G-F^ *N* = 8, *p* = 0.0006) for WT (gray bars) and *App*^NL-G-F^ (red bars) showing a strong degradation in *App*^NL-G-F^ mice from 2 m.o. onwards. **d** Summary of normalized frequency variance at 1 m.o. (*p* = 0.7254), 2 m.o. (*p* = 0.036), 4 m.o. (*p* = 0.0102) and 6 m.o. (*p* = 0.0051) for WT (gray bars) and *App*^NL-G-F^ (red bars) showing loss of regularity of gamma oscillations in *App*^NL-G-F^ from 2 m.o. onwards. **e** Representative power spectra from WT (gray lines) and *App*^NL-G-F^ (red lines), at the different ages analyzed. Data in bar graphs are presented as mean ± SEM. “*N*” indicates the number of mice. Statistics from two-way ANOVA followed by a Holm–Sidak’s multiple comparisons test (Supplementary Table 1). *p < 0.05, **p < 0.01, ***p < 0.001.

Our results show similar gamma oscillation power (*p* = 0.9724; Fig. 1b, c) and rhythm regularity as evidenced by frequency variance (*p* = 0.7254; Fig. 1d, e) between the *App*^NL-G-F^ mice and the WT group at 1 m.o. Interestingly, at 2 m.o. we found a significant decrease in gamma oscillation power (*p* = 0.0006; Fig. 1b, c) and significant increase of rhythm irregularity (*p* = 0.036; Fig. 1d, e) compared to age-matched WT animals. At 2 m.o. no Aβ plaque formation nor cognitive impairment, but a significant increase in Aβ concentration is reported in *App*^NL-G-F^ mice [30–32]. Consistent with the early functional disruption at 2 m.o., at 4 m.o. we found a significant reduction of gamma oscillation power (*p* = 0.0006; Fig. 1b, c) and an increasingly disordered rhythm as evidenced by a further increase in frequency variance (*p* = 0.0102; Fig. 1d, e) compared to the age-matched WT animals. *App*^NL-G-F^ mice at 4 m.o show a significant Aβ plaque load in the cortex and hippocampus, whereas cognitive impairment remains absent [30, 31]. For the last time point (6 m.o.), for which a robust Aβ plaque load as well as cognitive impairment is reported in *App*^NL-G-F^ mice [30–32], we found a significant decrease of gamma oscillation power (*p* = 0.0006; Fig. 1b, c) and an increase in frequency variance (*p* = 0.0051; Fig. 1d, e).

Taken together our first data set shows an early and severe decrease of gamma oscillation power with levels depressing further over development of the disease, and an early loss of gamma oscillation regularity. This suggests that neuronal classes involved in setting the gamma oscillation rhythm are compromised already at 2 months of age. In addition, our data corroborate our findings from the acute AD model of Aβ-induced loss of gamma oscillation power and rhythmicity [4, 21].

### Decrease of excitatory input to FSNs in App^NL-G-F^ mice

The balance between excitation and inhibition is essential for neuronal synchronization and network rhythmicity in gamma oscillations [10]. Studies have shown that the pathological accumulation of Aβ in AD affects the excitatory transmission onto inhibitory FSN [27, 37], the neuronal class that is majorly responsible for setting the gamma oscillation rhythm. Here we selected the three different disease stages that showed a reduction in the gamma oscillation analysis (2, 4 and 6 m.o.), to evaluate postsynaptic excitatory transmission onto FSN. For this purpose, we performed whole-cell voltage-clamp recordings before (quiescent network state) and after 30 min of KA (100 nM, activated network state).

**Fig. 2:**
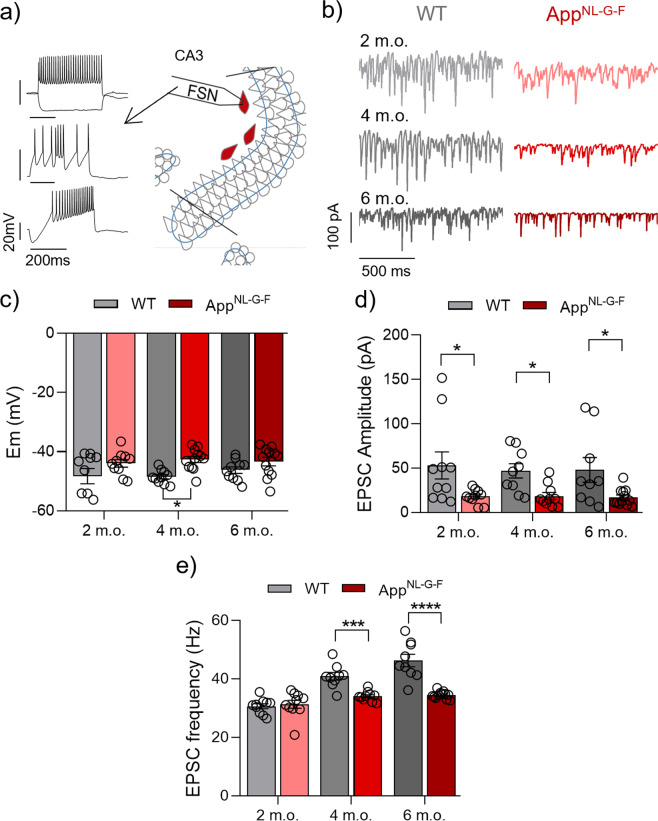
Decrease of excitatory input to FSNs in *App*^NL-G-F^ mice in the activated network state (KA present). **a** Representative diagram of FSN location (right side) in area CA3 of hippocampal slices and representative traces of FSN characteristic firing (left side) from a two-voltage step protocol (top), burst-eliciting protocol (middle) and voltage ramp protocol (bottom). **b** Representative sample traces of EPSC recordings from WT (left side) and *App*^NL-G-F^ (right side) at 2 m.o., 4 m.o. and 6 m.o. **c** Summary of FSN Em data at 2 m.o. (WT *n*:10 (*N* = 7) vs *App*^NL-G-F^ *n*:10 (*N* = 8), *p* = 0.0942), 4 m.o. (WT *n*:9 (*N* = 6) vs *App*^NL-G-F^ *n*: 12 (*N* = 7), *p* = 0.0152) and 6 m.o. (WT *n*:10 (*N* = 6) vs *App*^NL-G-F^ *n*: 12 (*N* = 7), *p* = 0.1654) for WT (gray bars) and *App*^NL-G-F^ (red bars). **d** Summary of EPSC amplitude data at 2 m.o. (WT *n*:6 (*N* = 7) vs *App*^NL-G-F^ *n*:7 (*N* = 8), *p* = 0.0222), 4 m.o. (WT *n*:9 (*N* = 6) vs *App*^NL-G-F^ *n*:10 (*N* = 7), *p* = 0.0331) and 6 m.o. (WT *n*:9 (*N* = 6) vs *App*^NL-G-F^ *n*:12 (*N* = 7), *p* = 0.0309) for WT (gray bars) and *App*^NL-G-F^ (red bars). **e** Summary of EPSC frequency data at 2 m.o. (*p* = 0.6769), 4 m.o. (*p* = 0.0003) and 6 m.o. (*p* < 0.0001) for WT (gray bars) and *App*^NL-G-F^ (red bars). Data in bar graphs are presented as mean ± SEM. “*n*” indicates the number of cells. “*N*” indicates the number of mice. Statistics from two-way ANOVA followed by a Holm–Sidak’s multiple comparisons test (Supplementary Table 1). *p < 0.05, ***p < 0.001, ****p < 0.0001.

Our results from FSN recordings in the quiescent state show no changes in membrane potential (Supplementary Fig. 1b), EPSC amplitude (Supplementary Fig. 1c) and EPSC frequency (Supplementary Fig. 1d) at any age. The activated state induced by KA provides us a tool to evaluate the cellular response to depolarization through glutamate receptor activation [38]. After KA-induced network activation, *App*^NL-G-F^ FSN show a decrease in the excitatory input at the three ages studied (Fig. 2b, d, e) and a significant increase in the depolarization at 4 m.o. compared to the WT group (*p* = 0.0152; Fig. 2c). The EPSC amplitude in the *App*^NL-G-F^ group at 2 m.o. shows a significant decrease compared to WT (*p* = 0.0222; Fig. 2d). Moreover, decrease in the EPSC amplitude in the *App*^NL-G-F^ group remains constant through the different animal ages investigated and shows a significant difference at 4 m.o. (*p* = 0.0331) and 6 m.o. (*p* = 0.0309; Fig. 2d). Interestingly, we found an increase in EPSC frequency in WT at 6 m.o.. This result could suggest synaptic adaptation aimed at maintaining the level of excitatory input to FSN necessary to keep the firing rate and synchrony of these neurons at normal levels (Figs. 2e and 4, respectively). In addition, we found no changes in the EPSC amplitude (Fig. 2d), the vector length and the firing rate (Fig. 4c, d) in FSN at 6 m.o. compared with the 2 m.o. and 4 m.o. within the WT group.

Altogether these results suggest a direct impact of the amyloidogenic pathology on the excitatory input to FSN. Diverse studies have shown that one of the features in AD is the functional disruption of inhibitory interneurons, mainly through impairment in their excitability [6, 22, 27].

### Decrease of excitatory and inhibitory input to PCs in App^NL-G-F^ mice

**Fig. 3:**
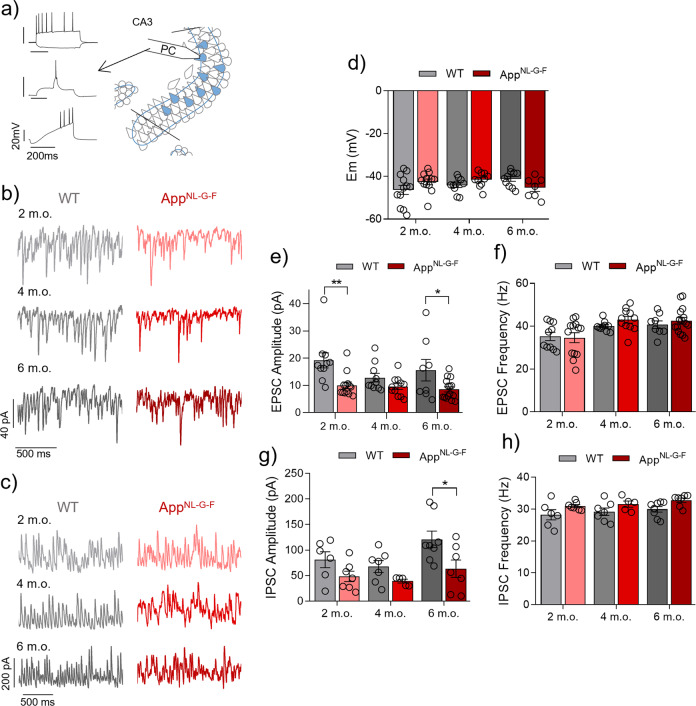
Decrease of excitatory and inhibitory input to PCs in *App*^NL-G-F^ mice in the activated network state (KA present). **a** Representative diagram of PC location (right side) in area CA3 of hippocampal slices and representative traces of PC characteristic firing (left side) from a two-voltage step protocol (top), burst-eliciting protocol (middle) and voltage ramp protocol (bottom). **b–c** Representative sample traces of (**b**) EPSC and (**c**) IPSC recordings from WT (left side) and *App*^NL-G-F^ (right side) at 2 m.o., 4 m.o. and 6 m.o. **d** Summary of PC Em data at 2 m.o. (WT *n*:10 (*N* = 8) vs *App*^NL-G-F^ *n*:12 (*N* = 8), *p* = 0.0.542), 4 m.o. (WT *n*:10 (*N* = 8) vs *App*^NL-G-F^ *n*:10 (*N* = 8), *p* = 0.2128) and 6 m.o. (WT *n*:10 (*N* = 6) vs *App*^NL-G-F^ *n*:7 (*N* = 8), *p* = 0.1538) for WT (gray bars) and *App*^NL-G-F^ (red bars). **e** Summary of EPSC amplitude data at 2 m.o. (WT *n*:10 (*N* = 8) vs *App*^NL-G-F^ *n*:13 (*N* = 8), *p* = 0.0025), 4 m.o. (WT *n*:10 (*N* = 7) vs *App*^NL-G-F^ *n*:11 (*N* = 8), *p* = 0.2284) and 6 m.o. (WT *n*:8 (*N* = 6) vs *App*^NL-G-F^ *n*:15 (*N* = 8), *p* = 0.0236) for WT (gray bars) and *App*^NL-G-F^ (red bars). **f** Summary of EPSC frequency data at 2 m.o (*p* = 0.8111), 4 m.o. (*p* = 0.5900) and 6 m.o. (*p* = 0.7432) for WT (gray bars) and *App*^NL-G-F^ (red bars). **g** Summary of IPSC amplitude data at 2 m.o. (WT *n*:6 (*N* = 6) vs *App*^NL-G-F^ *n*:7 (*N* = 6), *p* = 0.2115), 4 m.o. (WT *n*:7 (*N* = 6) vs *App*^NL-G-F^ *n*:5 (*N* = 5), *p* = 0.2115) and 6 m.o. (WT *n*:8 (*N* = 6) vs *App*^NL-G-F^ n:7 (*N* = 6), *p* = 0.0123) for WT (gray bars) and *App*^NL-G-F^ (red bars). **h** Summary of IPSC frequency data at 2 m.o (*p* = 0.1284), 4 m.o. (*p* = 0.1284) and 6 m.o. (*p* = 0.0289) for WT (gray bars) and *App*^NL-G-F^ (red bars). Data in bar graphs are presented as mean ± SEM. “*n*” indicates the number of cells. “*N*” indicates the number of mice. Statistics from two-way ANOVA followed by a Holm–Sidak’s multiple comparisons test (Supplementary Table 1). *p < 0.05, **p < 0.01.

Next, we evaluated the excitatory input to PC in the quiescent and activated network states. In the quiescent configuration, we found a significant decrease of EPSC amplitude (Supplementary Fig. 2c) and frequency (Supplementary Fig. 2d) for the *App*^NL-G-F^ group compared to WT at all three age points investigated with neuronal Em remaining unaffected (Supplementary Fig. 2b). This information suggests a synaptic dysregulation that corresponds well with previous reports in which the pathological concentration of Aβ could affect the glutamatergic transmission by internalization of AMPA receptors [39]. In the activated state of PC, the KA addition caused a similar depolarization of PC in both groups at 2, 4 and 6 m.o. (Fig. 3d). Moreover, the excitatory input onto PCs in the *App*^NL-G-F^ group shows a decrease when compared with the WT group at 2 m.o. (*p* = 0.0025) and 6 m.o. (*p* = 0.0236) as indicated by the EPSC amplitude (Fig. 3e), whereas EPSC frequency (Fig. 3f) remains unaffected. The results at 4 m.o. show no significant effect on the EPSC amplitude between groups (*p* = 0.1582).

Gamma oscillations induced by KA depend, apart from the excitatory glutamatergic transmission, on the inhibitory GABAergic transmission onto PCs [33]. This GABAergic input is mainly provided by FSN [7, 10, 11, 22]. Taking all this into account we assessed the inhibitory input to PC. Our results in the *App*^*NL-G-F*^ group show a not significant decrease in IPSC amplitude at 2 m.o. (*p* = 0.2115) and 4 m.o. (*p* = 0.2115), and a significant decrease at 6 m.o. (*p* = 0.0123) when compared with the age-matched WT groups (Fig. 3g). The IPSC frequency shows a non-significant increase in the *App*^NL-G-F^ group at 2 m.o. (*p* = 0.1284), 4 m.o. (*p* = 0.1284), and 6 m.o. (*p* = 0.1183) compared with the WT group (Fig. 3h). Our results evidence an alteration of the PC inhibitory input that most likely originates from FSNs. This supports the hypothesis that the pathological accumulation of Aβ causes a dysfunction of the activity of these inhibitory interneurons [40].

### Impaired FSN spike-gamma coupling in App^NL-G-F^ mice appears at 2 months of age

**Fig. 4:**
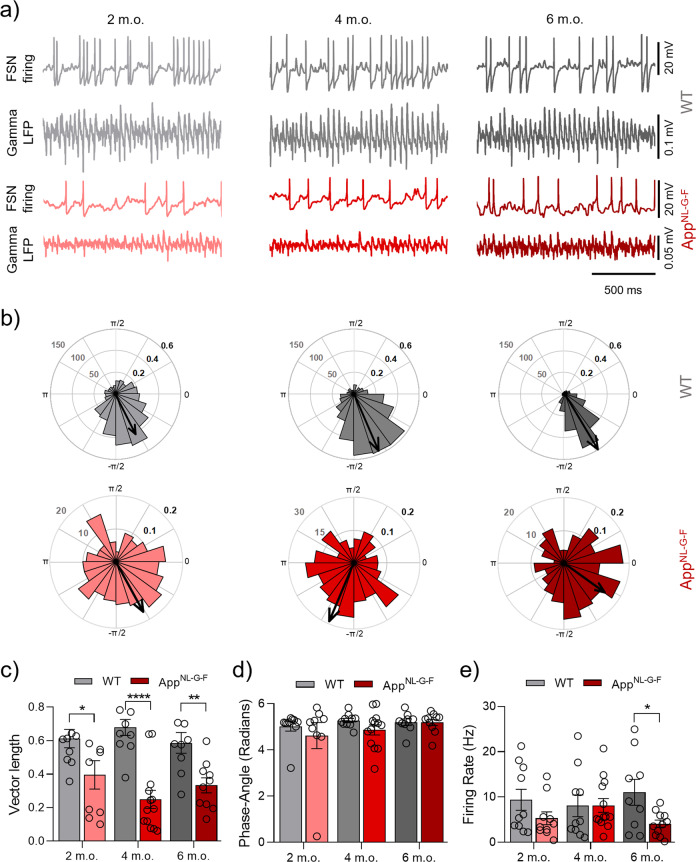
Impaired FSN spike-gamma coupling in *App*^NL-G-F^ mice appears at 2 m.o. (KA present). **a** Representative traces from concomitant recordings of FSN firing and LFP gamma oscillations at 2 m.o., 4 m.o. and 6 m.o. from WT (gray) and *App*^NL-G-F^ (red). **b** Representative polar plots of a representative FSN AP-phase angle at 2 m.o., 4 m.o. and 6 m.o. from WT (gray) and *App*^NL-G-F^ (red). **c** Summary of FSN vector length data at 2 m.o. (WT *n*:10 (*N* = 7) vs *App*^NL-G-F^ *n*:10 (*N* = 7), *p* = 0.0088), 4 m.o. (WT *n*:10 (*N* = 7) vs *App*^NL-G-F^ *n*:13 (*N* = 7), *p* < 0.0001) and 6 m.o. (WT *n*:9 (*N* = 6) vs *App*^NL-G-F^ *n*:10 (*N* = 7), *p* = 0.0082) for WT (gray bars) and *App*^NL-G-F^ (red bars). **d** Summary of FSN phase angle data at 2 m.o. (*p* = 0.4241), 4 m.o. (*p* = 0.4472) and 6 m.o. (*p* = 0.9844) for WT (gray bars) and *App*^NL-G-F^ (red bars). **e** Summary of FSN firing rate data at 2 m.o. (*p* = 0.2775), 4 m.o. (*p* = 0.9762) and 6 m.o. (*p* = 0.0479) for WT (gray bars) and *App*^NL-G-F^ (red bars). “*n*” indicates the number of cells. “*N*” indicates the number of mice. Data in bar graphs are presented as mean ± SEM. Statistics from two-way ANOVA followed by a Holm–Sidak’s multiple comparisons test (Supplementary Table 1). *p < 0.05, **p < 0.01, ****p < 0.0001.

In our previous work, we have shown that Aβ acutely impairs the spike-gamma coupling of PCs and FSNs leading to degradation of gamma oscillations in WT mice [4, 21, 41]. Until now it has remained an open question whether spike-gamma coupling of FSN or PC is progressively affected in response to chronically elevated Aβ42 levels in vivo such as in the *App*^NL-G-F^ AD model. To investigate this, we performed concomitant whole-cell current-clamp recordings of FSN APs during ongoing gamma oscillations recorded as LFP. Our results show a significant decrease in the spike-gamma coupling of FSN (vector length; Fig. 4c) in the *App*^NL-G-F^ group at the three age points: at 2 (*p* = 0.0088), 4 (*p* < 0.0001) and 6 m.o. (*p* = 0.0082 Fig. 4c). No changes were found for the phase angle at the three age points (Fig. 4d). The firing rate showed no changes in the *App*^NL-G-F^ group compared to the WT group at 2 m.o. (*p* = 0.2775), at 4 m.o. (*p* = 0.9762) and a significant reduction at 6 m.o. (*p* = 0.0479; Fig. 4e). Altogether these findings show how the increased level of Aβ_1-42_ reported by Saito et al. in 2014 impairs the spike-gamma coupling of FSNs very early on at 2 m.o. before the onset of Aβ plaque formation. This result indicates that the spike-gamma coupling capability from the FSN gets impaired during the Aβ amyloidosis progression. Since evidence published during the last years has shown the crucial role of inhibitory FSNs in gamma oscillation generation and maintenance [22, 25, 34, 41], the impaired FSN spike-gamma coupling that we found correlates with, and could potentially explain, the early and progressive deterioration of gamma oscillation power. To further validate that the Aβ amyloidosis causes the spike-gamma coupling impairment we evaluated single-cell patch clamp recordings of FSN at 1 m.o. in the *App*^*NL-G-F*^ mice. We found no changes in the vector length (*p* = 0.5542), phase angle (*p* = 0.6345) and firing rate (*p* = 0.9968) from the *App*^*NL-G-F*^ mice compared to age-matched WT mice (Supplementary Fig. 3).

### Impaired PC spike-gamma coupling in App^NL-G-F^ mice appears at 6 months of age

**Fig. 5:**
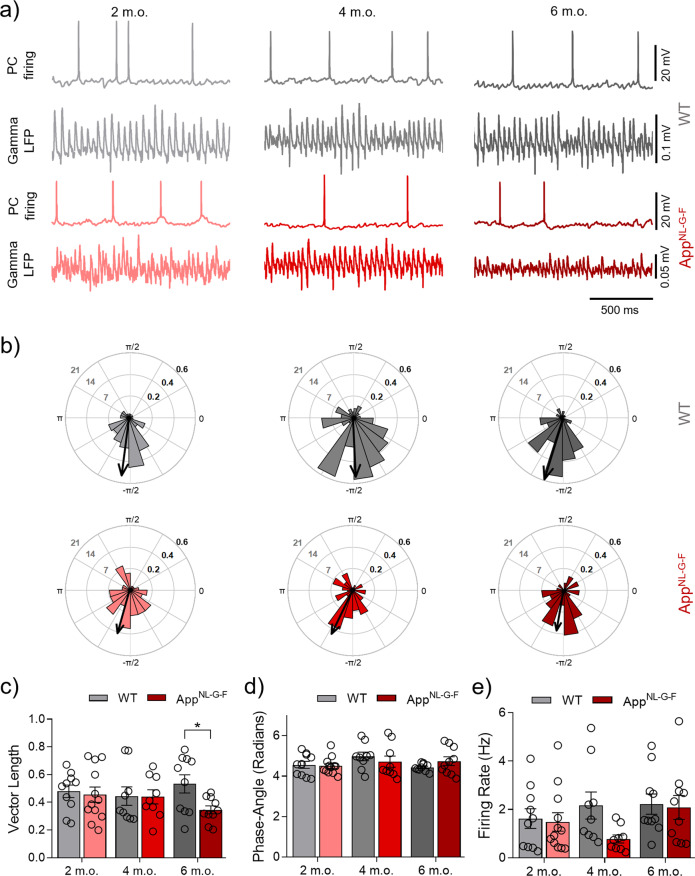
Impaired PC spike-gamma coupling in *App*^NL-G-F^ mice appears at 6 m.o. (KA present). **a** Representative traces from concomitant recordings of PC firing and LFP gamma oscillations at 2 m.o., 4 m.o. and 6 m.o. from WT (gray) and *App*^NL-G-F^ (red). **b** Representative polar plots of a representative PC AP-phase angle at 2 m.o., 4 m.o. and 6 m.o. from WT (gray) and *App*^NL-G-F^ (red). **c** Summary of PC vector length data at 2 m.o. (WT *n*:10 (*N* = 8) vs *App*^NL-G-F^ *n*:12 (*N* = 8), *p* = 0.9313), 4 m.o. (WT *n*:9 (*N* = 6) vs *App*^NL-G-F^ *n*:9 (*N* = 8), *p* = 0.9522) and 6 m.o. (WT *n*:10 (*N* = 6) vs *App*^NL-G-F^ *n*:10 (*N* = 8), *p* = 0.0491) for WT (gray bars) and *App*^NL-G-F^ (red bars). **d** Summary of PC phase angle data at 2 m.o. (*p* = 0.8264), 4 m.o. (*p* = 0.5664) and 6 m.o. (*p* = 0.5456) for WT (gray bars) and *App*^NL-G-F^ (red bars). **e** Summary of PC firing rate data at 2 m.o. (*p* = 0.9646), 4 m.o. (*p* = 0.0943) and 6 m.o. (*p* = 0.9646) for WT (gray bars) and *App*^NL-G-F^ (red bars). Data in bar graphs are presented as mean ± SEM. “*n*” indicates the number of cells. “*N*” indicates the number of mice. Statistics from two-way ANOVA followed by a Holm–Sidak’s multiple comparisons test (Supplementary Table 1). *p < 0.05.

To complete the analysis of the two major neuronal components contributing to network gamma oscillations we investigated the spike-gamma coupling of PCs using concomitant recordings as described above for FSN. Our results show no changes in the PC spike-gamma coupling (vector length; Fig. 5c) from the *App*^NL-G-F^ group at the two younger ages: at 2 m.o. (*p* = 0.9313) and at 4 m.o. (*p* = 0.9522). However, at 6 m.o. we found a significant reduction in the *App*^NL-G-F^ group vs the WT group (*p* = 0.0491; Fig. 5c). As for FSN, the AP phase angle remained unchanged at all age points investigated (Fig. 5d). AP firing rate showed no significant changes in the *App*^NL-G-F^ group at 2 m.o. (*p* = 0.9646), 4 m.o. (*p* = 0.0943) and 6 m.o. (*p* = 0.9646; Fig. 5e). It is possible that the firing rate results provide a window into an ongoing compensatory process within the PC population vs disease progression drivers. Particularly, the non-significant decrease of the firing rate at 4 m.o. could conceivably be linked to the emergence of Aβ plaques in the brains of *App*^NL-G-F^ mice at this age [30]. However, even when the firing rate is affected the resultant spike-gamma coupling remains similar to the WT group at this age (Fig. 5c). This could suggest that PC, despite the temporary sag in AP frequency, are able to maintain their cognition-relevant function during this process. Such possible compensatory mechanisms in the network are overtaken at 6 m.o., when the pathology is fully established [30] and results in loss of spike-gamma coupling in PCs in comparison to the impaired spike-gamma coupling in FSN. It is interesting to note that impaired spike-gamma coupling in PCs arises only at 6 m.o., matching the time point at which impairment of cognitive functions appears in the *App*^NL-G-F^ mice [30, 31].

In addition, we evaluated the AP waveforms from FSNs and PCs to test for a potential deterioration of AP shape. Whereas results from FSN recordings evidenced no changes in the AP waveform (Supplementary Fig. 4a), we found a reduction in the AP half-width in PC at 4 m.o. and 6 m.o. (Supplementary Fig. 4b). This may suggest that the AP waveform in PCs becomes affected after the onset of Aβ plaques [30]. Moreover, these timing of these results coincides with the emerging difference in the K^+^ inward current recorded in PCs at 6 m.o. (Supplementary Fig. 2).

## Discussion

In this study, we investigated the deterioration of cognition-relevant hippocampal network gamma oscillations and the changes of the activity of the responsible neuronal classes in the novel *App*^NL-G-F^ AD mouse model. We described the changes in the oscillating network as well as in FSNs and PCs across the progression of the pathology in the *App*^*NL-G-F*^ knock-in mice at 1, 2, 4 and 6-months of age compared to age-matched WT mice. Our results demonstrate that the degradation of gamma oscillations far precedes, and so is independent of, the formation of Aβ plaques and observable cognitive decline [30–32]. We suggest that the gamma oscillation impairment found at 2 m.o. (but not at 1 m.o.) is due to the mounting effect of increased levels of Aβ_42_ reported in the *App*^*NL-G-F*^ mouse model [30–32].

We show an aggressive disruption of hippocampal network gamma oscillations at 2 m.o. in the *App*^NL-G-F^ mouse model. Also, we provide evidence that the impaired spike-gamma coupling of inhibitory FSN is responsible for the impairment of gamma oscillations from 2 m.o. onwards, whereas the spike-gamma coupling of excitatory PC remains unaffected until 6 m.o., when the onset of cognitive impairment is reported [30–32]. Importantly, the results of the present study using an AD mouse model (chronic AD model) support the conclusions we derived from our earlier work using an acute ex vivo Aβ AD model (acute AD model) regarding the role of impaired FSN (and PC) spike-gamma coupling for the degradation of cognition-relevant gamma oscillations [4, 17].

Gamma oscillations are present in the hippocampal formation, a brain structure that plays an essential role in cognition and memory formation [7, 10, 42]. This insight extends to AD patients, where a progressive loss of gamma-band synchronization occurs in lock-step with the progressive loss of cognitive functions typical of the disease [7–9]. In the past, different approaches have been used to understand whether loss of rhythmicity of cognition-relevant gamma oscillations (and the underlying neuronal synchronization) is correlated with the pathologic aggregation of Aβ in the brain, and if so, which one occurs first and might therefore be a good diagnostic tool and therapeutic target in the clinic.

We have previously demonstrated that Aβ degrades gamma oscillation in the hippocampus as well as theta oscillations in the medial septum [4, 21, 43]. In the hippocampus these changes are caused by impaired spike-gamma coupling in FSN and PC [4, 21, 41]. Other studies, using transgenic mouse models that overexpress APP found similar results [18, 22–26, 44]. However, these transgenic mouse models overexpress APP in a non-physiological, indiscriminate way, which is a major concern when it comes to interpreting data derived from these mouse models [45]. This artefact is circumvented in the App knock-in mice (*App*^NL-G-F^) free of APP overexpression and exhibiting a robust Aβ pathology [30, 31]. Recent reports indicate changes to brain oscillations during in vivo recordings in *App*^NL-G-F^ mice at 5 months of age in the entorhinal cortex [46] and 8 months of age in the prefrontal cortex [47]. These studies support the hypothesis of a correlation between gamma oscillations and the appearance of Aβ plaques, but provide no information about what happens in the very early stages of the pathology when therapeutic intervention would be most promising.

We found that *App*^NL-G-F^ mice exhibit a significant degradation of in vitro gamma oscillation power as early as 2 months of age (Fig.1b, c). Evidence suggests that the pathological increase of Aβ down-regulates the expression of synaptic receptors such as GluA4 [22, 27] or Nav1.1 [18]. This downregulation of excitatory receptors/channels could explain the decrease in excitatory input onto the FSN at the three ages investigated (Fig. 2d, e). The shifted balance between excitation and inhibition likely causes the impaired spike-gamma coupling of FSN action potentials, occurring at the latest at the same time point as the gamma oscillation degradation (Fig. 4c).

The shifted equilibrium between excitation and inhibition in the network could either underlie or reflect the impaired spike-gamma coupling of both FSN and PC. Here, we observed severe disturbances in the excitatory drive onto both neuronal classes during disease progression. This disturbance was asymmetrically patterned with the excitatory drive onto FSN diminishing at 4 m.o and onto PC at 2 m.o. Finally, at 6 m.o the brake-down of excitatory drive onto FSN and PC converges with a decrease of IPSC amplitudes at higher frequencies compared to WT counterparts, which represents the collapse of synaptic transmission in the hippocampal network of *App*^NL-G-F^ mice. Collectively this could indicate a dramatic loss of neuronal synaptic ability to maintain proper communication as well as network performance and stability necessary for information processing. However, more experiments are needed to determine whether FSN dysfunction is causally responsible for the impairment of PC function in the amyloidogenic pathology progression.

In parallel, biomarkers of neuroinflammatory response such as GFAP and IBA1 are unchanged at 2 m.o. (Supplementary Fig. 5a, b). This means that the synaptic dysfunction evidenced by the lack of synchrony and reduced gamma oscillation power is not a consequence of microgliosis or astrogliosis, but a direct effect of Aβ on neuronal physiology. This is not in conflict with the possibility that inflammation might worsen the pathology in the brain later in the disease progression, which has been suggested in previous characterizations of the mouse model [30, 31]. Our results suggest to us a new paradigm: the pathological concentration of soluble Aβ species, prior to any fibrillization and aggregation into plaques, disrupts gamma network rhythmicity by impairing the spike-gamma coupling of FSN activity, and triggers the loss of neuronal homeostasis long before the onset of cognitive impairment.

Recent evidence has shown the dysfunction in inhibitory interneurons before the onset of Aβ plaques but with an inherent dependency on the cognitive impairment already established in different AD mouse models [18, 24–26, 29]. However, our findings using the *App*^NL-G-F^ mouse model at 2 m.o. demonstrate a specific dysfunction of inhibitory FSN during functional network gamma oscillations (Fig. 4c) whereas the resting membrane potential and EPSC are not affected in quiescent state (Supplementary Fig. 1b). Particularly, this result highlights the value of timely detection of network dysfunction related to inhibitory FSN in the amyloidogenic pathology development. We support this paradigm with current and previous findings from our laboratory as well as in others, reporting the rescue of gamma oscillations by the manipulation of inhibitory interneurons which causes an improvement of the possible treatments of the Aβ pathology [18, 24, 28, 41]. Further experiments need to be done to demonstrate the early gamma oscillation disruption in the *App*^NL-G-F^ mouse model in vivo and to correlate such mouse data with human clinical data.

## Conclusions

In summary, our data shows that impaired FSN spike-gamma coupling is the earliest functional impairment in the amyloidogenic pathology progression and could be the main cause for gamma oscillation degradation and cognitive impairment. Therefore, we hypothesize that the early detection and restoration of impaired spike-gamma coupling in FSN in prodromal AD patients might prevent the consequent degradation of gamma oscillations and subsequent cognitive impairment. Based on pre-clinical and clinical data these changes to neuronal activity and their effect on cognition-relevant brain rhythms are functional biomarkers in AD and correlate better with cognitive decline than traditional expression biomarkers such as Aβ plaque load. Also, these changes in neuronal activity and impairment of gamma oscillations are present already in the very early stages of the pathology and are more likely to be amenable to rescue than any restoration attempts in later, post-plaque, stages of the pathology. In conclusion, our findings provide evidence of an Aβ-induced mechanism responsible for the earliest disruption of cognition-relevant gamma oscillations in AD and suggest an alternative therapeutic approach.

## Supplementary information

Supplementary information

Supplementary Figure 1.

Supplementary Figure 2.

Supplementary Figure 3.

Supplementary Figure 4.

Supplementary Figure 5.

## Data Availability

Raw data are available from the corresponding authors upon reasonable request.
