# Supplementary material for: Impaired spike-gamma coupling of area CA3 fast-spiking interneurons as the earliest functional impairment in the AppNL-G-F mouse model of Alzheimer’s disease
Source: Mol Psychiatry. 2021 Aug 12;26(10):5557–67. doi: 10.1038/s41380-021-01257-0 (PMC8758494; doi:10.1038/s41380-021-01257-0)
Supplement: Supplementary file 1 — Supplementary information [file 41380_2021_1257_MOESM1_ESM.docx]

**Title: Impaired spike-gamma coupling of area CA3 fast-spiking interneurons as the earliest functional impairment in the *App^NL-G-F^* mouse model of Alzheimer’s Disease**

**Authors:** Luis Enrique Arroyo-García^a^*, Arturo G. Isla^a^, Yuniesky Andrade-Talavera^a^, Hugo Balleza-Tapia^a^, Raúl Loera-Valencia^b^, Laura Alvarez-Jimenez^b^, Giusy Pizzirusso^a^, Simone Tambaro^b^, Per Nilsson^b^ & André Fisahn^a^*

**Affiliation:** *^a^ Neuronal Oscillations Laboratory; ^a,b^ Division of Neurogeriatrics; Center for Alzheimer Research; Dept. of Neurobiology, Care Sciences and Society; Karolinska Institutet, 17164 Solna, Sweden*

**corresponding authors*

**Supplementary information**

**Supplementary Figure 1. Electrophysiological characterization of FSN in the quiescent network state (without KA).**

**a)** Representative sample traces of EPSC recordings in quiescent state from WT (left side) and *App*^NL-G-F^ (right side) at 2 m.o., 4 m.o. and 6 m.o. **b)** Summary of FSN Em data at 2 m.o. (WT n:6 (N=6) vs *App*^NL-G-F^ n:11 (N=8), p=0.9080), 4 m.o. (WT n:6 (N=6) vs *App*^NL-G-F^ n:9 (N=7), p=0.9080) and 6 m.o. (WT n:8 (N=6) vs *App*^NL-G-F^ n:9 (N=6), p=0.9080) for WT (grey bars) and *App*^NL-G-F^ (red bars). **c)** Summary of EPSC amplitude data at 2 m.o. (WT n:6 (N=6) vs *App*^NL-G-F^ n:11 (N=8), p=0.6413), 4 m.o. (WT n:6 (N=6) vs *App*^NL-G-F^ n:9 (N=7), p=0.6413) and 6 m.o. (WT n:8 (N=6) vs *App*^NL-G-F^ n:9 (N=6), p=0.6413) for WT (grey bars) and *App*^NL-G-F^ (red bars). **d)**. Summary of EPSC frequency data at 2 m.o. (p=0.4176), 4 m.o. (p=0.8849) and 6 m.o. (p=0.3366) for WT (grey bars) and *App*^NL-G-F^ (red bars). **e)** Summary of EPSC charge transfer data at 2 m.o. (p=0.5271), 4 m.o. (p=0.8679) and 6 m.o. (p=0.4239) for WT (grey bars) and *App*^NL-G-F^ (red bars). **f)** I-V summary response at 2 m.o. (WT n:8 (N=6) vs *App*^NL-G-F^ n:6 (N=6), Two-way ANOVA test: F (1, 180) = 7.907, p= 0.0055), 4 m.o. (WT n:8 (N=6) vs *App*^NL-G-F^ n:6 (N=6), Two-way ANOVA test: F (1, 180) = 5.553, p= 0.0195) and 6 m.o. (WT n:7 (N=6) vs *App*^NL-G-F^ n:9 (N=6), Two-way ANOVA test: F (1, 210) = 42.23, p< 0.0001) for WT (grey bars) and *App*^NL-G-F^ (red bars). **g)** Representative I-V traces at 2 m.o., 4 m.o. and 6 m.o. from WT (grey) and *App*^NL-G-F^ (red). Data in bar graphs is presented as mean ± SEM. “n” indicates the number of cells. “N” indicates the number of mice. Statistics from two-way ANOVA followed by a Holm-Sidak's multiple comparisons test (Supp. Table 1). *p<0.05, **p<0.01, ****p<0.0001.

**Supplementary Figure 2. Electrophysiological characterization of PC in the quiescent network state (without KA).**

**a)** Representative sample traces of EPSC recordings in quiescent state from WT (left side) and *App*^NL-G-F^ (right side) at 2 m.o., 4 m.o. and 6 m.o. **b)** Summary of PC Em data at 2 m.o. (WT n:6 (N=6) vs *App*^NL-G-F^ n:6 (N=6), p=0.2583), 4 m.o. (WT n:8 (N=6) vs *App*^NL-G-F^ n:6 (N=6), p=0.8982) and 6 m.o. (WT n:8 (N=6) vs *App*^NL-G-F^ n:9 (N=7), p=0.9726) for WT (grey bars) and *App*^NL-G-F^ (red bars). **c)** Summary of EPSC amplitude data at 2 m.o. (WT n:6 (N=6) vs *App*^NL-G-F^ n:6 (N=6), p=0.0015), 4 m.o. (WT n:8 (N=6) vs *App*^NL-G-F^ n:7 (N=6), p=0.0407) and 6 m.o. (WT n:8 (N=6) vs *App*^NL-G-F^ n:9 (N=7), p=0.0407) for WT (grey bars) and *App*^NL-G-F^ (red bars). **d)**. Summary of EPSC frequency data at 2 m.o. (p=0.0017), 4 m.o. (p=0.0369) and 6 m.o. (p=0.0017) for WT (grey bars) and *App*^NL-G-F^ (red bars). **e)** Summary of EPSC charge transfer data at 2 m.o. (p=0.0048), 4 m.o. (p=0.0185) and 6 m.o. (p= 0.0185) for WT (grey bars) and *App*^NL-G-F^ (red bars). **f)** I-V summary response at 2 m.o. (WT n:8 (N=6) vs *App*^NL-G-F^ n:6 (N=6), Two-way ANOVA test: F (1, 210) = 3.295, p= 0.0709), 4 m.o. (WT n:8 (N=6) vs *App*^NL-G-F^ n:6 (N=6), Two-way ANOVA test: F (1, 180) = 16.24, p< 0.0001) and 6 m.o. (WT n:7 (N=6) vs *App*^NL-G-F^ n:9 (N=7), Two-way ANOVA test: F (1, 165) = 47.23, p< 0.0001) for WT (grey bars) and *App*^NL-G-F^ (red bars). **g)** Representative I-V traces at 2 m.o., 4 m.o. and 6 m.o. from WT (grey) and *App*^NL-G-F^ (red). Data in bar graphs is presented as mean ± SEM. “n” indicates the number of cells. “N” indicates the number of mice. Statistics from two-way ANOVA followed by a Holm-Sidak's multiple comparisons test (Supp. Table 1). *p<0.05, **p<0.01, ****p<0.0001.

**Supplementary Figure 3. FSN spike-gamma coupling in *App^NL-G-F^* mice at 1 m.o. is unaffected during KA-induced gamma oscillations.**

**a**) Summary of FSN vector length data at 1 m.o. (WT n:9 (N=4) vs *App^NL-G-F^* n:11 (N=5), t-test: p= 0.5542). **b**) Summary of FSN phase angle data (t-test: p= 0.6345). **c**) Summary of FSN firing rate data (t-test: p= 0.9968), for WT (grey bars) and *App^NL-G-F^* (red bars). “n” indicates the number of cells. “N” indicates the number of mice. Data in bar graphs is presented as mean ± SEM. t-test: Student's t-test.

**Supplementary Figure 4. Decrease of PC action potential half-width in *App^NL-G-F^* mice at 4 m.o..**

**A.** Analysis of action potential half-width vs firing frequency from FSN at 2, 4 and 6 m.o. for WT (grey) and *App^NL-G-F^* (red). **a)** Summary of FSN action potential half-width vs firing frequency data at 2 m.o. (Half-width: WT n:7 (N=6) vs *App^NL-G-F^* n:7 (N=6), t-test: p= 0.3269; Firing frequency: t-test: p= 0.0558), **b)** at 4 m.o. (Half-width: WT n:7 (N=6) vs *App^NL-G-F^* n:13 (N=7), MW test: p= 0.8773; Firing frequency: t-test: p= 0.6782) and **c)** at 6 m.o. (Half-width: WT n:9 (N=6) vs *App^NL-G-F^* n:14 (N=7), t-test: p= 0.3624; Firing frequency: t-test: p= 0.0201). **B.** Analysis of action potential half-width vs firing frequency from PC at 2, 4 and 6 m.o. for WT (grey) and *App^NL-G-F^* (red). **a)** Summary of PC action potential half-width vs firing frequency data at 2 m.o. (Half-width: WT n:7 (N=7) vs *App^NL-G-F^* n:5 (N=5255), t-test: p= 0.3269; Firing frequency: t-test: p= 0.9617), **b)** at 4 m.o. (Half-width: WT n:10 (N=6) vs *App^NL-G-F^* n:10 (N=8), t-test: p= 0.0181; Firing frequency: MW test: p= 0.0455) and **c)** at 6 m.o. (Half-width: WT n:8 (N=6) vs *App^NL-G-F^* n:8 (N=8), t-test: p= 0.0065; Firing frequency: MW test: p= 0.5935). “n” indicates the number of cells. “N” indicates the number of mice. Data in bar graphs is presented as mean ± SEM. MW test: Mann Whitney test, t-test: Student's t-test. *p<0.05, **p<0.01.

**Supplementary Figure 5. Neuroinflammation in *App*^NL-G-F^ mice is evident at 6 m.o.**

**a)** Summary graphs of the normalized GFAP expression levels at 2 m.o. (WT N=3 vs *App*^NL-G-F^ N=3, p=0.) and 6 m.o. (WT n: 6 vs *App*^NL-G-F^ N=5, t-test: p= 0.0126) for WT (white) and *App*^NL-G-F^ (red). **b)** Summary graphs of the normalized IBA1 expression levels at 2 m.o. (WT N=3 vs *App*^NL-G-F^ N=3, MW test: p= 0.2507) and 6 m.o. (WT N=6 vs *App*^NL-G-F^ N=5, t-test: p= 0.0073) for WT (white) and *App*^NL-G-F^ (red). **c)** Representative western blots of WT and *App*^NL-G-F^ cortex-hippocampus preparations at 2 m.o. and 6 m.o. against the inflammatory marker GFAP. **d)** Representative western blots of WT and *App*^NL-G-F^ cortex-hippocampus preparations at 2 m.o. and 6 m.o. against the inflammatory marker IBA-1. Data in bar graphs is presented as mean ± SEM from animal determination. “N” indicates the number of mice. Statistics from two-way ANOVA followed by a Holm-Sidak's multiple comparisons test (Supp. Table 1). *p<0.05, **p<0.01, ****p<0.0001.

**Supplementary table 1. Graph data**

| **Figure 1** | | | | | | |  |  |  |  |
| --- | --- | --- | --- | --- | --- | --- | --- | --- | --- | --- |
|  |  |  |  |  |  |  |  | Source of variation | Test | P value |
| **Panel c** | Gamma Power (normalized) | | | | | | Two-way ANOVA test | Column factor | F (1, 48) = 32.04 | P<0.0001 |
|  | WT | | | App^NL-G-F^ | | | Holm-Sidak's multiple comparisons test | | Power (1β err p) |  |
| Age | Mean | SD | n | Mean | SD | n | Significant | P value |  |  |
| 1 m.o. | 100 | 29.267 | 5 | 99.146 | 36.371 | 4 | No | 0.9724 | 0.053718 |  |
| 2 m.o. | 100 | 62.289 | 9 | 30.395 | 30.998 | 10 | Yes | 0.0006 | 0.904623 |  |
| 4 m.o. | 100 | 41.120 | 6 | 20.106 | 11.348 | 8 | Yes | 0.0006 | 0.998469 |  |
| 6 m.o. | 100 | 34.704 | 6 | 19.444 | 15.698 | 8 | Yes | 0.0006 | 0.999809 |  |
|  |  |  |  |  |  |  |  |  |  |  |
|  |  |  |  |  |  |  |  | Source of variation | Test | P value |
| **Panel d** | Frequency variance (normalized) | | | | | | Two-way ANOVA test | Column factor | F (1, 48) = 15.55 | P=0.0003 |
|  | WT | | | App^NL-G-F^ | | | Holm-Sidak's multiple comparisons test | |  |  |
| Age | Mean | SD | n | Mean | SD | n | Significant | P value |  |  |
| 1 m.o. | 100 | 37.073 | 5 | 91.912 | 13.088 | 4 | No | 0.7254 |  |  |
| 2 m.o. | 100 | 34.732 | 9 | 138.345 | 14.068 | 10 | Yes | 0.036 |  |  |
| 4 m.o. | 100 | 25.904 | 6 | 156.748 | 29.875 | 8 | Yes | 0.0102 |  |  |
| 6 m.o. | 100 | 22.021 | 6 | 163.066 | 61.425 | 8 | Yes | 0.0051 |  |  |
|  |  |  |  |  |  |  |  |  |  |  |
| **Figure 2** | | | | | | |  |  |  |  |
|  |  |  |  |  |  |  |  |  |  |  |
|  |  |  |  |  |  |  |  | Source of variation | Test | P value |
| **Panel c** | Em (mV) | | | | | | Two-way ANOVA test | Column factor | F (1, 57) = 13.46 | P=0.0005 |
|  | WT | | | App^NL-G-F^ | | | Holm-Sidak's multiple comparisons test | |  |  |
| Age | Mean | SD | n | Mean | SD | n | Significant | P value |  |  |
| 2 m.o. | -48.249 | 8.047 | 10 | -43.974 | 3.984 | 10 | No | 0.0942 |  |  |
| 4 m.o. | -48.730 | 2.220 | 9 | -42.644 | 3.561 | 12 | Yes | 0.0152 |  |  |
| 6 m.o. | -46.222 | 3.445 | 10 | -43.372 | 4.934 | 12 | No | 0.1654 |  |  |
|  |  |  |  |  |  |  |  |  |  |  |
|  |  |  |  |  |  |  |  | Source of variation | Test | P value |
| **Panel d** | EPSC Amplitude (pA) | | | | | | Two-way ANOVA test | Column factor | F (1, 54) = 18.53 | P<0.0001 |
|  | WT | | | App^NL-G-F^ | | | Holm-Sidak's multiple comparisons test | |  |  |
| Age | Mean | SD | n | Mean | SD | n | Significant | P value |  |  |
| 2 m.o. | 53.090 | 48.244 | 10 | 18.117 | 8.135 | 10 | Yes | 0.0222 |  |  |
| 4 m.o. | 46.837 | 24.310 | 9 | 18.588 | 12.732 | 10 | Yes | 0.0331 |  |  |
| 6 m.o. | 47.969 | 41.818 | 9 | 16.995 | 9.456 | 12 | Yes | 0.0309 |  |  |
|  |  |  |  |  |  |  |  |  |  |  |
|  |  |  |  |  |  |  |  | Source of variation | Test | P value |
| **Panel e** | EPSC Frequency (Hz) | | | | | | Two-way ANOVA test | Column factor | F (1, 54) = 39.90 | P<0.0001 |
|  | WT | | | App^NL-G-F^ | | | Holm-Sidak's multiple comparisons test | |  |  |
| Age | Mean | SD | n | Mean | SD | n | Significant | P value |  |  |
| 2 m.o. | 30.637 | 2.635 | 10 | 31.322 | 4.330 | 10 | No | 0.6769 |  |  |
| 4 m.o. | 40.907 | 3.918 | 9 | 34.058 | 1.681 | 10 | Yes | 0.0003 |  |  |
| 6 m.o. | 46.272 | 6.384 | 9 | 34.467 | 1.215 | 12 | Yes | <0.0001 |  |  |
|  |  |  |  |  |  |  |  |  |  |  |
| **Figure 3** | | | | | | |  |  |  |  |
|  |  |  |  |  |  |  |  |  |  |  |
|  |  |  |  |  |  |  |  | Source of variation | Test | P value |
| **Panel d** | Em (mV) | | | | | | Two-way ANOVA test | Column factor | F (1, 53) = 0.8423 | P=0.3629 |
|  | WT | | | App^NL-G-F^ | | | Holm-Sidak's multiple comparisons test | |  |  |
| Age | Mean | SD | n | Mean | SD | n | Significant | P value |  |  |
| 2 m.o. | -47.466 | 7.157 | 10 | -42.586 | 4.606 | 12 | No | 0.0542 |  |  |
| 4 m.o. | -44.140 | 3.507 | 10 | -41.498 | 3.407 | 10 | No | 0.2128 |  |  |
| 6 m.o. | -41.194 | 3.623 | 10 | -45.313 | 4.753 | 7 | No | 0.1538 |  |  |
|  |  |  |  |  |  |  |  |  |  |  |
|  |  |  |  |  |  |  |  | Source of variation | Test | P value |
| **Panel e** | EPSC Amplitude (pA) | | | | | | Two-way ANOVA test | Column factor | F (1, 61) = 17.70 | P<0.0001 |
|  | WT | | | App^NL-G-F^ | | | Holm-Sidak's multiple comparisons test | |  |  |
| Age | Mean | SD | n | Mean | SD | n | Significant | P value |  |  |
| 2 m.o. | 19.199 | 8.709 | 10 | 10.040 | 4.320 | 13 | Yes | 0.0025 |  |  |
| 4 m.o. | 12.856 | 5.077 | 10 | 9.566 | 3.607 | 11 | No | 0.2284 |  |  |
| 6 m.o. | 15.581 | 11.167 | 8 | 8.554 | 3.732 | 15 | Yes | 0.0236 |  |  |
|  |  |  |  |  |  |  |  |  |  |  |
|  |  |  |  |  |  |  |  | Significant | Test | P value |
| **Panel f** | EPSC frequency (Hz) | | | | | | Two-way ANOVA test | Column factor | F (1, 61) = 0.8774 | P=0.3526 |
|  | WT | | | App^NL-G-F^ | | | Holm-Sidak's multiple comparisons test | |  |  |
| Age | Mean | SD | n | Mean | SD | n | Significant | P value |  |  |
| 2 m.o. | 35.255 | 6.096 | 10 | 34.651 | 8.364 | 13 | No | 0.8111 |  |  |
| 4 m.o. | 40.032 | 2.272 | 10 | 43.020 | 5.188 | 11 | No | 0.59 |  |  |
| 6 m.o. | 40.727 | 5.095 | 8 | 42.531 | 6.026 | 15 | No | 0.7432 |  |  |
|  |  |  |  |  |  |  |  |  |  |  |
|  |  |  |  |  |  |  |  | Source of variation | Test | P value |
| **Panel g** | IPSC Amplitude (pA) | | | | | | Two-way ANOVA test | Column factor | F (1, 34) = 11.77 | P=0.0016 |
|  | WT | | | App^NL-G-F^ | | | Holm-Sidak's multiple comparisons test | |  |  |
| Age | Mean | SD | n | Mean | SD | n | Significant | P value |  |  |
| 2 m.o. | 81.073 | 38.420 | 6 | 48.305 | 27.002 | 7 | No | 0.2115 |  |  |
| 4 m.o. | 67.837 | 30.534 | 7 | 39.267 | 8.043 | 5 | No | 0.2115 |  |  |
| 6 m.o. | 120.967 | 45.572 | 8 | 63.500 | 45.024 | 7 | Yes | 0.0123 |  |  |
|  |  |  |  |  |  |  |  |  |  |  |
|  |  |  |  |  |  |  |  | Source of variation | Test | P value |
| **Panel h** | IPSC frequency (Hz) | | | | | | Two-way ANOVA test | Column factor | F (1, 34) = 10.34 | P=0.0029 |
|  | WT | | | App^NL-G-F^ | | | Holm-Sidak's multiple comparisons test | |  |  |
| Age | Mean | SD | n | Mean | SD | n | Significant | P value |  |  |
| 2 m.o. | 28.222 | 3.934 | 6 | 30.943 | 1.237 | 7 | No | 0.1284 |  |  |
| 4 m.o. | 29.169 | 3.095 | 7 | 31.570 | 2.185 | 5 | No | 0.1284 |  |  |
| 6 m.o. | 29.933 | 2.462 | 8 | 32.767 | 1.848 | 7 | No | 0.1183 |  |  |
|  |  |  |  |  |  |  |  |  |  |  |
| **Figure 4** | | | | | | |  |  |  |  |
|  |  |  |  |  |  |  |  |  |  |  |
|  |  |  |  |  |  |  |  | Source of variation | Test | P value |
| **Panel c** | Vector Length | | | | | | Two-way ANOVA test | Column factor | F (1, 56) = 41.18 | P<0.0001 |
|  | WT | | | App^NL-G-F^ | | | Holm-Sidak's multiple comparisons test | | Power (1β err p) |  |
| Age | Mean | SD | n | Mean | SD | n | Significant | P value |  |  |
| 2 m.o. | 0.612 | 0.172 | 10 | 0.389 | 0.240 | 10 | Yes | 0.0088 | 0.742854 |  |
| 4 m.o. | 0.679 | 0.151 | 10 | 0.249 | 0.194 | 13 | Yes | <0.0001 | 0.999972 |  |
| 6 m.o. | 0.586 | 0.187 | 9 | 0.333 | 0.142 | 10 | Yes | 0.0082 | 0.937655 |  |
|  |  |  |  |  |  |  |  |  |  |  |
|  |  |  |  |  |  |  |  | Source of variation | Test | P value |
| **Panel d** | Phase-Angle (Radians) | | | | | | Two-way ANOVA test | Column factor | F (1, 56) = 2.117 | P=0.1512 |
|  | WT | | | App^NL-G-F^ | | | Holm-Sidak's multiple comparisons test | |  |  |
| Age | Mean | SD | n | Mean | SD | n | Significant | P value |  |  |
| 2 m.o. | 5.017 | 0.638 | 10 | 4.492 | 1.645 | 10 | No | 0.4241 |  |  |
| 4 m.o. | 5.276 | 0.286 | 10 | 4.871 | 0.796 | 13 | No | 0.4472 |  |  |
| 6 m.o. | 5.202 | 0.425 | 9 | 5.194 | 0.439 | 10 | No | 0.9844 |  |  |
|  |  |  |  |  |  |  |  |  |  |  |
|  |  |  |  |  |  |  |  | Source of variation | Test | P value |
| **Panel e** | Firing rate (Hz) | | | | | | Two-way ANOVA test | Column factor | F (1, 57) = 5.311 | P=0.0249 |
|  | WT | | | App^NL-G-F^ | | | Holm-Sidak's multiple comparisons test | |  |  |
| Age | Mean | SD | n | Mean | SD | n | Significant | P value |  |  |
| 2 m.o. | 9.367 | 7.330 | 10 | 5.302 | 4.504 | 10 | No | 0.2775 |  |  |
| 4 m.o. | 8.065 | 7.583 | 10 | 8.144 | 5.540 | 13 | No | 0.9762 |  |  |
| 6 m.o. | 11.044 | 8.581 | 9 | 4.108 | 2.661 | 11 | Yes | 0.0479 |  |  |
|  |  |  |  |  |  |  |  |  |  |  |
| **Figure 5** | | | | | | |  |  |  |  |
|  |  |  |  |  |  |  |  |  |  |  |
|  |  |  |  |  |  |  |  | Source of variation | Test | P value |
| **Panel c** | Vector Length | | | | | | Two-way ANOVA test | Column factor | F (1, 54) = 2.696 | P=0.1064 |
|  | WT | | | App^NL-G-F^ | | | Holm-Sidak's multiple comparisons test | | Power (1β err p) |  |
| Age | Mean | SD | n | Mean | SD | n | Significant | P value |  |  |
| 2 m.o. | 0.480 | 0.142 | 10 | 0.455 | 0.192 | 12 | No | 0.9313 | 0.094990 |  |
| 4 m.o. | 0.445 | 0.200 | 9 | 0.440 | 0.153 | 9 | No | 0.9522 | 0.056171 |  |
| 6 m.o. | 0.533 | 0.207 | 10 | 0.345 | 0.091 | 10 | Yes | 0.0491 | 0.811518 |  |
|  |  |  |  |  |  |  |  |  |  |  |
|  |  |  |  |  |  |  |  | Source of variation | Test | P value |
| **Panel d** | Phase-Angle (Radians) | | | | | | Two-way ANOVA test | Column factor | F (1, 54) = 4.444e-005 | P=0.9947 |
|  | WT | | | App^NL-G-F^ | | | Holm-Sidak's multiple comparisons test | |  |  |
| Age | Mean | SD | n | Mean | SD | n | Significant | P value |  |  |
| 2 m.o. | 4.561 | 0.547 | 10 | 4.507 | 0.418 | 12 | No | 0.8264 |  |  |
| 4 m.o. | 4.976 | 0.639 | 9 | 4.716 | 0.835 | 9 | No | 0.5664 |  |  |
| 6 m.o. | 4.434 | 0.184 | 10 | 4.745 | 0.671 | 10 | No | 0.5456 |  |  |
|  |  |  |  |  |  |  |  |  |  |  |
|  |  |  |  |  |  |  |  | Source of variation | Test | P value |
| **Panel e** | Firing rate (Hz) | | | | | | Two-way ANOVA test | Column factor | F (1, 54) = 2.536 | P=0.1171 |
|  | WT | | | App^NL-G-F^ | | | Holm-Sidak's multiple comparisons test | |  |  |
| Age | Mean | SD | n | Mean | SD | n | Significant | P value |  |  |
| 2 m.o. | 1.622 | 1.286 | 10 | 1.485 | 1.316 | 12 | No | 0.9646 |  |  |
| 4 m.o. | 2.163 | 1.677 | 9 | 0.779 | 0.498 | 9 | No | 0.0943 |  |  |
| 6 m.o. | 2.220 | 1.318 | 10 | 2.083 | 1.582 | 10 | No | 0.9646 |  |  |
|  |  |  |  |  |  |  |  |  |  |  |
|  |  |  |  |  |  |  |  |  |  |  |
| **Supplementary figure 1** | | | | | | |  |  |  |  |
|  |  |  |  |  |  |  |  |  |  |  |
|  |  |  |  |  |  |  |  | Source of variation | Test | P value |
| **Panel b** | Em (mV) | | | | | | Two-way ANOVA test | Row Factor | F (2, 43) = 3.411 | P=0.0422 |
|  | WT | | | App^NL-G-F^ | | | Holm-Sidak's multiple comparisons test | |  |  |
| Age | Mean | SD | n | Mean | SD | n | Significant | P value |  |  |
| 2 m.o. | -51.706 | 11.061 | 6 | -49.250 | 5.417 | 11 | No | 0.908 |  |  |
| 4 m.o. | -52.428 | 7.797 | 6 | -50.653 | 5.542 | 9 | No | 0.908 |  |  |
| 6 m.o. | -57.750 | 10.256 | 8 | -56.813 | 8.380 | 9 | No | 0.908 |  |  |
|  |  |  |  |  |  |  |  |  |  |  |
|  |  |  |  |  |  |  |  | Source of variation | Test | P value |
| **Panel c** | EPSC Amplitude (pA) | | | | | | Two-way ANOVA test | None |  |  |
|  | WT | | | App^NL-G-F^ | | | Holm-Sidak's multiple comparisons test | |  |  |
| Age | Mean | SD | n | Mean | SD | n | Significant | P value |  |  |
| 2 m.o. | 12.102 | 4.067 | 6 | 10.136 | 4.038 | 11 | No | 0.6413 |  |  |
| 4 m.o. | 9.776 | 3.503 | 6 | 11.202 | 5.553 | 9 | No | 0.6413 |  |  |
| 6 m.o. | 9.438 | 3.295 | 8 | 7.377 | 2.327 | 9 | No | 0.6413 |  |  |
|  |  |  |  |  |  |  |  |  |  |  |
|  |  |  |  |  |  |  |  | Source of variation | Test | P value |
| **Panel d** | EPSC Frequency (Hz) | | | | | | Two-way ANOVA test | None |  |  |
|  | WT | | | App^NL-G-F^ | | | Holm-Sidak's multiple comparisons test | |  |  |
| Age | Mean | SD | n | Mean | SD | n | Significant | P value |  |  |
| 2 m.o. | 10.247 | 3.459 | 6 | 8.389 | 3.480 | 11 | No | 0.4176 |  |  |
| 4 m.o. | 8.664 | 1.232 | 6 | 8.430 | 2.666 | 9 | No | 0.8849 |  |  |
| 6 m.o. | 8.552 | 2.721 | 8 | 6.250 | 3.591 | 9 | No | 0.3366 |  |  |
|  |  |  |  |  |  |  |  |  |  |  |
|  |  |  |  |  |  |  |  | Source of variation | Test | P value |
| **Panel e** | EPSC Charge transfer (pC) | | | | | | Two-way ANOVA test | None |  |  |
|  | WT | | | App^NL-G-F^ | | | Holm-Sidak's multiple comparisons test | |  |  |
| Age | Mean | SD | n | Mean | SD | n | Significant | P value |  |  |
| 2 m.o. | 71.242 | 30.906 | 6 | 54.227 | 39.801 | 11 | No | 0.5271 |  |  |
| 4 m.o. | 55.120 | 28.443 | 6 | 58.012 | 36.872 | 9 | No | 0.8679 |  |  |
| 6 m.o. | 50.893 | 29.454 | 8 | 28.543 | 24.066 | 9 | No | 0.4239 |  |  |
|  |  |  |  |  |  |  |  |  |  |  |
| **Panel f** | Current desnity | | | | | |  |  |  |  |
|  |  |  |  |  |  |  |  |  |  |  |
| **Age** | **2 m.o.** |  |  |  |  |  |  |  |  |  |
|  |  |  |  |  |  |  |  |  |  |  |
|  | WT | | | App^NL-G-F^ | | |  |  |  |  |
| Voltage | Mean | SD | n | Mean | SD | n | Adjusted P value (Two-way ANOVA) |  |  |  |
| -90.000 | -2.181 | 1.328 | 8 | -2.413 | 2.225 | 6 | >0.9999 |  |  |  |
| -80.000 | -1.182 | 0.860 | 8 | -1.020 | 0.756 | 6 | >0.9999 |  |  |  |
| -70.000 | -0.127 | 0.309 | 8 | 0.178 | 0.363 | 6 | >0.9999 |  |  |  |
| -60.000 | 0.846 | 0.362 | 8 | 1.047 | 0.873 | 6 | >0.9999 |  |  |  |
| -50.000 | 1.890 | 0.805 | 8 | 2.208 | 1.693 | 6 | >0.9999 |  |  |  |
| -40.000 | 3.055 | 1.155 | 8 | 4.145 | 3.240 | 6 | >0.9999 |  |  |  |
| -30.000 | 4.958 | 1.684 | 8 | 7.396 | 5.590 | 6 | >0.9999 |  |  |  |
| -20.000 | 7.706 | 2.655 | 8 | 11.986 | 8.363 | 6 | >0.9999 |  |  |  |
| -10.000 | 11.883 | 4.532 | 8 | 17.853 | 11.666 | 6 | 0.9993 |  |  |  |
| 0.000 | 17.228 | 7.275 | 8 | 24.104 | 14.844 | 6 | 0.9964 |  |  |  |
| 10.000 | 22.742 | 9.751 | 8 | 31.108 | 18.232 | 6 | 0.976 |  |  |  |
| 20.000 | 29.087 | 12.629 | 8 | 38.679 | 21.482 | 6 | 0.9269 |  |  |  |
| 30.000 | 35.895 | 16.026 | 8 | 46.596 | 24.969 | 6 | 0.846 |  |  |  |
| 40.000 | 43.005 | 19.172 | 8 | 54.357 | 27.776 | 6 | 0.7827 |  |  |  |
| 50.000 | 50.691 | 22.886 | 8 | 63.322 | 30.873 | 6 | 0.6342 |  |  |  |
|  |  |  |  |  |  |  |  |  |  |  |
| **Age** | **4 m.o.** |  |  |  |  |  |  |  |  |  |
|  |  |  |  |  |  |  |  |  |  |  |
|  | WT | | | App^NL-G-F^ | | |  |  |  |  |
| Voltage | Mean | SD | n | Mean | SD | n | Adjusted P value (Two-way ANOVA) |  |  |  |
| -90.000 | -2.603 | 0.995 | 8 | -2.918 | 1.299 | 6 | >0.9999 |  |  |  |
| -80.000 | -1.659 | 0.767 | 8 | -1.431 | 0.640 | 6 | >0.9999 |  |  |  |
| -70.000 | -0.671 | 0.832 | 8 | -0.039 | 0.095 | 6 | >0.9999 |  |  |  |
| -60.000 | 0.355 | 1.103 | 8 | 1.388 | 0.574 | 6 | >0.9999 |  |  |  |
| -50.000 | 1.499 | 1.616 | 8 | 3.215 | 1.452 | 6 | >0.9999 |  |  |  |
| -40.000 | 3.102 | 2.439 | 8 | 6.210 | 3.006 | 6 | >0.9999 |  |  |  |
| -30.000 | 6.542 | 5.325 | 8 | 11.263 | 6.014 | 6 | >0.9999 |  |  |  |
| -20.000 | 12.541 | 10.342 | 8 | 18.886 | 10.049 | 6 | >0.9999 |  |  |  |
| -10.000 | 20.840 | 16.868 | 8 | 28.778 | 14.908 | 6 | >0.9999 |  |  |  |
| 0.000 | 30.440 | 23.775 | 8 | 40.781 | 20.412 | 6 | >0.9999 |  |  |  |
| 10.000 | 41.265 | 31.939 | 8 | 54.087 | 26.389 | 6 | 0.9996 |  |  |  |
| 20.000 | 53.090 | 40.727 | 8 | 69.168 | 33.215 | 6 | 0.9949 |  |  |  |
| 30.000 | 64.690 | 49.609 | 8 | 85.024 | 40.400 | 6 | 0.9558 |  |  |  |
| 40.000 | 76.729 | 57.945 | 8 | 101.824 | 47.994 | 6 | 0.8088 |  |  |  |
| 50.000 | 89.167 | 66.815 | 8 | 119.419 | 55.807 | 6 | 0.5416 |  |  |  |
|  |  |  |  |  |  |  |  |  |  |  |
| **Age** | **4 m.o.** |  |  |  |  |  |  |  |  |  |
|  |  |  |  |  |  |  |  |  |  |  |
|  | WT | | | App^NL-G-F^ | | |  |  |  |  |
| Voltage | Mean | SD | n | Mean | SD | n | Adjusted P value (Two-way ANOVA) |  |  |  |
| -90.000 | -1.182 | 0.115 | 7 | -3.335 | 0.321 | 9 | >0.9999 |  |  |  |
| -80.000 | -0.586 | 0.059 | 7 | -1.451 | 0.134 | 9 | >0.9999 |  |  |  |
| -70.000 | -0.027 | 0.015 | 7 | 0.103 | 0.066 | 9 | >0.9999 |  |  |  |
| -60.000 | 0.515 | 0.042 | 7 | 1.532 | 0.230 | 9 | >0.9999 |  |  |  |
| -50.000 | 1.109 | 0.084 | 7 | 3.133 | 0.528 | 9 | >0.9999 |  |  |  |
| -40.000 | 2.029 | 0.113 | 7 | 5.636 | 1.063 | 9 | >0.9999 |  |  |  |
| -30.000 | 3.787 | 0.153 | 7 | 9.977 | 1.948 | 9 | >0.9999 |  |  |  |
| -20.000 | 6.835 | 0.620 | 7 | 16.328 | 3.123 | 9 | 0.9961 |  |  |  |
| -10.000 | 11.189 | 1.497 | 7 | 25.236 | 4.564 | 9 | 0.8814 |  |  |  |
| 0.000 | 16.546 | 2.527 | 7 | 35.410 | 6.184 | 9 | 0.4893 |  |  |  |
| 10.000 | 22.226 | 3.626 | 7 | 47.235 | 8.161 | 9 | 0.1101 |  |  |  |
| 20.000 | 28.730 | 4.996 | 7 | 59.238 | 10.159 | 9 | 0.0181 |  |  |  |
| 30.000 | 35.825 | 6.570 | 7 | 72.569 | 12.106 | 9 | 0.0016 |  |  |  |
| 40.000 | 43.592 | 8.366 | 7 | 85.557 | 14.023 | 9 | 0.0002 |  |  |  |
| 50.000 | 51.069 | 10.025 | 7 | 98.582 | 15.943 | 9 | <0.0001 |  |  |  |
|  |  |  |  |  |  |  |  |  |  |  |
| **Supplementary figure 2** | | | | | | |  |  |  |  |
|  |  |  |  |  |  |  |  |  |  |  |
|  |  |  |  |  |  |  |  | Source of variation | Test | P value |
| **Panel b** | Em (mV) | | | | | | Two-way ANOVA test | None |  |  |
|  | WT | | | App^NL-G-F^ | | | Holm-Sidak's multiple comparisons test | |  |  |
| Age | Mean | SD | n | Mean | SD | n | Significant | P value |  |  |
| 2 m.o. | -66.319 | 4.159 | 6 | -60.921 | 5.525 | 6 | No | 0.2583 |  |  |
| 4 m.o. | -66.822 | 5.407 | 8 | -65.601 | 3.929 | 6 | No | 0.8982 |  |  |
| 6 m.o. | -63.951 | 7.009 | 8 | -63.860 | 5.428 | 9 | No | 0.9726 |  |  |
|  |  |  |  |  |  |  |  |  |  |  |
|  |  |  |  |  |  |  |  | Source of variation | Test | P value |
| **Panel c** | EPSC amplitude (pA) | | | | | | Two-way ANOVA test | Column factor | F (1, 38) = 25.19 | P<0.0001 |
|  | WT | | | App^NL-G-F^ | | | Holm-Sidak's multiple comparisons test | |  |  |
| Age | Mean | SD | n | Mean | SD | n | Significant | P value |  |  |
| 2 m.o. | 15.238 | 3.615 | 6 | 7.165 | 0.887 | 6 | Yes | 0.0015 |  |  |
| 4 m.o. | 11.849 | 4.985 | 8 | 7.330 | 2.824 | 7 | Yes | 0.0407 |  |  |
| 6 m.o. | 12.672 | 5.252 | 8 | 8.345 | 1.988 | 9 | Yes | 0.0407 |  |  |
|  |  |  |  |  |  |  |  |  |  |  |
|  |  |  |  |  |  |  |  | Source of variation | Test | P value |
| **Panel d** | EPSC Frequency (Hz) | | | | | | Two-way ANOVA test | Column factor | F (1, 38) = 30.95 | P<0.0001 |
|  | WT | | | App^NL-G-F^ | | | Holm-Sidak's multiple comparisons test | |  |  |
| Age | Mean | SD | n | Mean | SD | n | Significant | P value |  |  |
| 2 m.o. | 11.367 | 1.068 | 6 | 6.617 | 1.928 | 6 | Yes | 0.0017 |  |  |
| 4 m.o. | 8.948 | 3.384 | 8 | 6.469 | 1.743 | 7 | Yes | 0.0369 |  |  |
| 6 m.o. | 9.383 | 2.464 | 8 | 5.341 | 1.625 | 9 | Yes | 0.0017 |  |  |
|  |  |  |  |  |  |  |  |  |  |  |
|  |  |  |  |  |  |  |  | Source of variation | Test | P value |
| **Panel** | EPSC Charge transfer (pC) | | | | | | Two-way ANOVA test | Column factor | F (1, 38) = 26.27 | P<0.0001 |
|  | WT | | | App^NL-G-F^ | | | Holm-Sidak's multiple comparisons test | |  |  |
| Age | Mean | SD | n | Mean | SD | n | Significant | P value |  |  |
| 2 m.o. | 136.925 | 25.216 | 6 | 53.635 | 26.267 | 6 | Yes | 0.0048 |  |  |
| 4 m.o. | 95.184 | 80.245 | 8 | 34.991 | 15.470 | 7 | Yes | 0.0185 |  |  |
| 6 m.o. | 87.091 | 45.930 | 8 | 31.512 | 8.272 | 9 | Yes | 0.0185 |  |  |
|  |  |  |  |  |  |  |  |  |  |  |
| **Panel f** | Current desnity | | | | | |  |  |  |  |
|  |  |  |  |  |  |  |  |  |  |  |
| **Age** | **2 m.o.** |  |  |  |  |  |  |  |  |  |
|  |  |  |  |  |  |  |  |  |  |  |
|  | WT | | | App^NL-G-F^ | | |  |  |  |  |
| Voltage | Mean | SD | n | Mean | SD | n | Adjusted P value (Two-way ANOVA) |  |  |  |
| -90.000 | -1.992 | 0.955 | 6 | -3.050 | 3.015 | 10 | >0.9999 |  |  |  |
| -80.000 | -1.024 | 0.506 | 6 | -1.474 | 1.459 | 10 | >0.9999 |  |  |  |
| -70.000 | -0.054 | 0.084 | 6 | -0.022 | 0.136 | 10 | >0.9999 |  |  |  |
| -60.000 | 0.901 | 0.516 | 6 | 1.526 | 1.894 | 10 | >0.9999 |  |  |  |
| -50.000 | 1.880 | 1.027 | 6 | 3.495 | 4.062 | 10 | >0.9999 |  |  |  |
| -40.000 | 3.019 | 1.637 | 6 | 6.045 | 6.917 | 10 | >0.9999 |  |  |  |
| -30.000 | 4.483 | 2.241 | 6 | 9.139 | 10.012 | 10 | >0.9999 |  |  |  |
| -20.000 | 6.631 | 3.533 | 6 | 12.990 | 13.647 | 10 | >0.9999 |  |  |  |
| -10.000 | 10.309 | 6.972 | 6 | 17.820 | 17.773 | 10 | >0.9999 |  |  |  |
| 0.000 | 16.142 | 12.362 | 6 | 24.935 | 23.094 | 10 | >0.9999 |  |  |  |
| 10.000 | 23.027 | 19.079 | 6 | 33.069 | 29.947 | 10 | >0.9999 |  |  |  |
| 20.000 | 30.646 | 26.037 | 6 | 42.038 | 37.799 | 10 | 0.9996 |  |  |  |
| 30.000 | 38.921 | 33.128 | 6 | 52.037 | 46.532 | 10 | 0.9981 |  |  |  |
| 40.000 | 47.453 | 40.239 | 6 | 62.171 | 55.410 | 10 | 0.9935 |  |  |  |
| 50.000 | 56.782 | 47.853 | 6 | 72.898 | 65.007 | 10 | 0.9842 |  |  |  |
|  |  |  |  |  |  |  |  |  |  |  |
| **Age** | **4 m.o.** |  |  |  |  |  |  |  |  |  |
|  |  |  |  |  |  |  |  |  |  |  |
|  | WT | | | App^NL-G-F^ | | |  |  |  |  |
| Voltage | Mean | SD | n | Mean | SD | n | Adjusted P value (Two-way ANOVA) |  |  |  |
| -90.000 | -1.623 | 1.652 | 8 | -4.064 | 6.001 | 6 | >0.9999 |  |  |  |
| -80.000 | -0.829 | 0.748 | 8 | -2.053 | 2.975 | 6 | >0.9999 |  |  |  |
| -70.000 | -0.038 | 0.310 | 8 | -0.046 | 0.169 | 6 | >0.9999 |  |  |  |
| -60.000 | 0.733 | 1.182 | 8 | 2.058 | 3.175 | 6 | >0.9999 |  |  |  |
| -50.000 | 1.643 | 2.170 | 8 | 4.183 | 6.308 | 6 | >0.9999 |  |  |  |
| -40.000 | 2.697 | 3.007 | 8 | 6.830 | 9.458 | 6 | >0.9999 |  |  |  |
| -30.000 | 4.033 | 3.932 | 8 | 10.318 | 13.015 | 6 | >0.9999 |  |  |  |
| -20.000 | 5.688 | 4.875 | 8 | 14.721 | 17.561 | 6 | 0.9996 |  |  |  |
| -10.000 | 7.802 | 5.697 | 8 | 20.321 | 22.269 | 6 | 0.986 |  |  |  |
| 0.000 | 10.967 | 6.226 | 8 | 26.701 | 27.783 | 6 | 0.9075 |  |  |  |
| 10.000 | 15.013 | 7.876 | 8 | 33.743 | 33.884 | 6 | 0.734 |  |  |  |
| 20.000 | 19.449 | 9.370 | 8 | 41.206 | 40.137 | 6 | 0.5017 |  |  |  |
| 30.000 | 24.771 | 11.403 | 8 | 49.256 | 46.704 | 6 | 0.311 |  |  |  |
| 40.000 | 30.356 | 13.515 | 8 | 57.183 | 52.966 | 6 | 0.1891 |  |  |  |
| 50.000 | 36.308 | 15.970 | 8 | 65.137 | 59.394 | 6 | 0.1172 |  |  |  |
|  |  |  |  |  |  |  |  |  |  |  |
| **Age** | **4 m.o.** |  |  |  |  |  |  |  |  |  |
|  |  |  |  |  |  |  |  |  |  |  |
|  | WT | | | App^NL-G-F^ | | |  |  |  |  |
| Voltage | Mean | SD | n | Mean | SD | n | Adjusted P value (Two-way ANOVA) |  |  |  |
| -90.000 | -1.290 | 0.203 | 6 | -2.777 | 0.814 | 7 | >0.9999 |  |  |  |
| -80.000 | -0.680 | 0.129 | 6 | -1.187 | 0.336 | 7 | >0.9999 |  |  |  |
| -70.000 | -0.162 | 0.062 | 6 | 0.056 | 0.029 | 7 | >0.9999 |  |  |  |
| -60.000 | 0.286 | 0.042 | 6 | 1.218 | 0.395 | 7 | >0.9999 |  |  |  |
| -50.000 | 0.777 | 0.055 | 6 | 2.761 | 0.904 | 7 | >0.9999 |  |  |  |
| -40.000 | 1.452 | 0.055 | 6 | 4.887 | 1.476 | 7 | >0.9999 |  |  |  |
| -30.000 | 2.441 | 0.117 | 6 | 8.480 | 2.519 | 7 | >0.9999 |  |  |  |
| -20.000 | 3.929 | 0.323 | 6 | 14.087 | 4.189 | 7 | 0.9992 |  |  |  |
| -10.000 | 5.958 | 0.694 | 6 | 22.058 | 6.467 | 7 | 0.9298 |  |  |  |
| 0.000 | 8.736 | 1.193 | 6 | 32.415 | 8.656 | 7 | 0.4623 |  |  |  |
| 10.000 | 12.477 | 1.927 | 6 | 44.246 | 11.140 | 7 | 0.0897 |  |  |  |
| 20.000 | 16.789 | 2.598 | 6 | 57.399 | 13.883 | 7 | 0.0077 |  |  |  |
| 30.000 | 21.851 | 3.544 | 6 | 70.853 | 16.565 | 7 | 0.0005 |  |  |  |
| 40.000 | 27.537 | 4.637 | 6 | 85.122 | 19.439 | 7 | <0.0001 |  |  |  |
| 50.000 | 33.366 | 5.648 | 6 | 99.099 | 22.354 | 7 | <0.0001 |  |  |  |
|  |  |  |  |  |  |  |  |  |  |  |
| **Supplementary figure 3** | | | | | | |  |  |  |  |
|  |  |  |  |  |  |  |  |  |  |  |
|  |  |  |  |  |  |  |  |  |  |  |
| **Panel a** | Vector lenght | | | | | |  |  |  |  |
|  | WT | | | App^NL-G-F^ | | | Unpaired t test |  |  |  |
| Age | Mean | SD | n | Mean | SD | n | Significant | P value |  |  |
| 1 m.o. | 0.742 | 0.208 | 9 | 0.6917 | 0.1623 | 11 | No | 0.5542 |  |  |
|  |  |  |  |  |  |  |  |  |  |  |
|  |  |  |  |  |  |  |  |  |  |  |
| **Panel b** | Phase-Angle (Radians) | | | | | |  |  |  |  |
|  | WT | | | App^NL-G-F^ | | | Unpaired t test |  |  |  |
| Age | Mean | SD | n | Mean | SD | n | Significant | P value |  |  |
| 1 m.o. | 4.791 | 0.285 | 9 | 4.883 | 0.5088 | 11 | No | 0.6345 |  |  |
|  |  |  |  |  |  |  |  |  |  |  |
|  |  |  |  |  |  |  |  |  |  |  |
| **Panel c** | Firing rate (Hz) | | | | | |  |  |  |  |
|  | WT | | | App^NL-G-F^ | | | Unpaired t test |  |  |  |
| Age | Mean | SD | n | Mean | SD | n | Significant | P value |  |  |
| 1 m.o. | 8.235 | 7.455 | 6 | 8.223 | 6.304 | 6 | No | 0.9968 |  |  |
|  |  |  |  |  |  |  |  |  |  |  |
| **Supplementary figure 4** | | | | | | |  |  |  |  |
|  |  |  |  |  |  |  |  |  |  |  |
|  |  |  |  |  |  |  |  |  |  |  |
| **Panel A** | FSN | | | | | |  |  |  |  |
| **Half-with** | WT | | | App^NL-G-F^ | | | Unpaired t test |  |  |  |
| Age | Mean | SD | n | Mean | SD | n | Significant | P value |  |  |
| 2 m.o. | 1.605 | 0.077 | 7 | 1.915 | 0.293 | 7 | No | 0.3269 |  |  |
| 4 m.o. | 1.924 | 0.173 | 7 | 2.094 | 0.215 | 13 | No | 0.8773 |  |  |
| 6 m.o. | 2.095 | 0.251 | 10 | 1.839 | 0.148 | 14 | No | 0.3624 |  |  |
|  |  |  |  |  |  |  |  |  |  |  |
|  |  | | | | | | Two-way ANOVA test | Column factor | F (1, 38) = 25.19 | P<0.0001 |
| **Frequency** | WT | | | App^NL-G-F^ | | | Holm-Sidak's multiple comparisons test | |  |  |
| Age | Mean | SD | n | Mean | SD | n | Significant | P value |  |  |
| 2 m.o. | 9.367 | 7.330 | 10 | 5.302 | 4.504 | 10 | No | 0.2775 |  |  |
| 4 m.o. | 8.065 | 7.583 | 10 | 8.144 | 5.540 | 13 | No | 0.9762 |  |  |
| 6 m.o. | 11.044 | 8.581 | 9 | 4.108 | 2.661 | 11 | Yes | 0.0479 |  |  |
|  |  |  |  |  |  |  |  |  |  |  |
|  |  |  |  |  |  |  |  |  |  |  |
| **Panel B** | PC | | | | | |  |  |  |  |
| **Half-with** | WT | | | App^NL-G-F^ | | | Unpaired t test |  |  |  |
| Age | Mean | SD | n | Mean | SD | n | Significant | P value |  |  |
| 2 m.o. | 2.691 | 0.212 | 8 | 2.509 | 0.068 | 5 | No | 0.3269 |  |  |
| 4 m.o. | 2.549 | 0.102 | 10 | 2.250 | 0.053 | 10 | Yes | 0.0181 |  |  |
| 6 m.o. | 2.961 | 0.202 | 8 | 2.258 | 0.087 | 8 | Yes | 0.0065 |  |  |
|  |  |  |  |  |  |  |  |  |  |  |
|  |  |  |  |  |  |  |  | Source of variation | Test | P value |
|  |  | | | | | | Two-way ANOVA test | Column factor | F (1, 54) = 2.536 | P=0.1171 |
| **Frequency** | WT | | | App^NL-G-F^ | | | Holm-Sidak's multiple comparisons test | |  |  |
| Age | Mean | SD | n | Mean | SD | n | Significant | P value |  |  |
| 2 m.o. | 1.622 | 1.286 | 10 | 1.485 | 1.316 | 12 | No | 0.9646 |  |  |
| 4 m.o. | 2.163 | 1.677 | 9 | 0.779 | 0.498 | 9 | No | 0.0943 |  |  |
| 6 m.o. | 2.220 | 1.318 | 10 | 2.083 | 1.582 | 10 | No | 0.9646 |  |  |
|  |  |  |  |  |  |  |  |  |  |  |
|  |  |  |  |  |  |  |  |  |  |  |
| **Supplementary figure 5** | | | | | | |  |  |  |  |
|  |  |  |  |  |  |  |  |  |  |  |
|  |  |  |  |  |  |  |  | Source of variation | Test | P value |
| **Panel a** | Normalized GFAP protein expression | | | | | | Two-way ANOVA test | None (col factor) | F (1, 13) = 4.605 | **P=0.0513** |
|  | WT | | | App^NL-G-F^ | | | Holm-Sidak's multiple comparisons test | |  |  |
| Age | Mean | SD | n | Mean | SD | n | Significant | P value |  |  |
| 2 m.o. | 100 | 51.750 | 3 | 173.120 | 98.631 | 3 | No | 0.1324 |  |  |
| 6 m.o. | 100 | 14.153 | 6 | 125.236 | 12.420 | 5 | No | 0.3722 |  |  |
|  |  |  |  |  |  |  |  |  |  |  |
|  |  |  |  |  |  |  |  | Source of variation | Test | P value |
| **Panel b** | Normalized IBA1 protein expression | | | | | | Two-way ANOVA test | Column factor | F (1, 15) = 8.700 | P=0.0099 |
|  | WT | | | App^NL-G-F^ | | | Holm-Sidak's multiple comparisons test | |  |  |
| Age | Mean | SD | n | Mean | SD | n | Significant | P value |  |  |
| 2 m.o. | 100 | 28.483 | 3 | 131.332 | 28.699 | 3 | No | 0.2057 |  |  |
| 6 m.o. | 100 | 20.244 | 6 | 153.206 | 34.888 | 7 | Yes | 0.0098 |  |  |
