# Supplementary figures and images for: Impaired spike-gamma coupling of area CA3 fast-spiking interneurons as the earliest functional impairment in the AppNL-G-F mouse model of Alzheimer’s disease
Source: Mol Psychiatry. 2021 Aug 12;26(10):5557–67. doi: 10.1038/s41380-021-01257-0 (PMC8758494; doi:10.1038/s41380-021-01257-0)

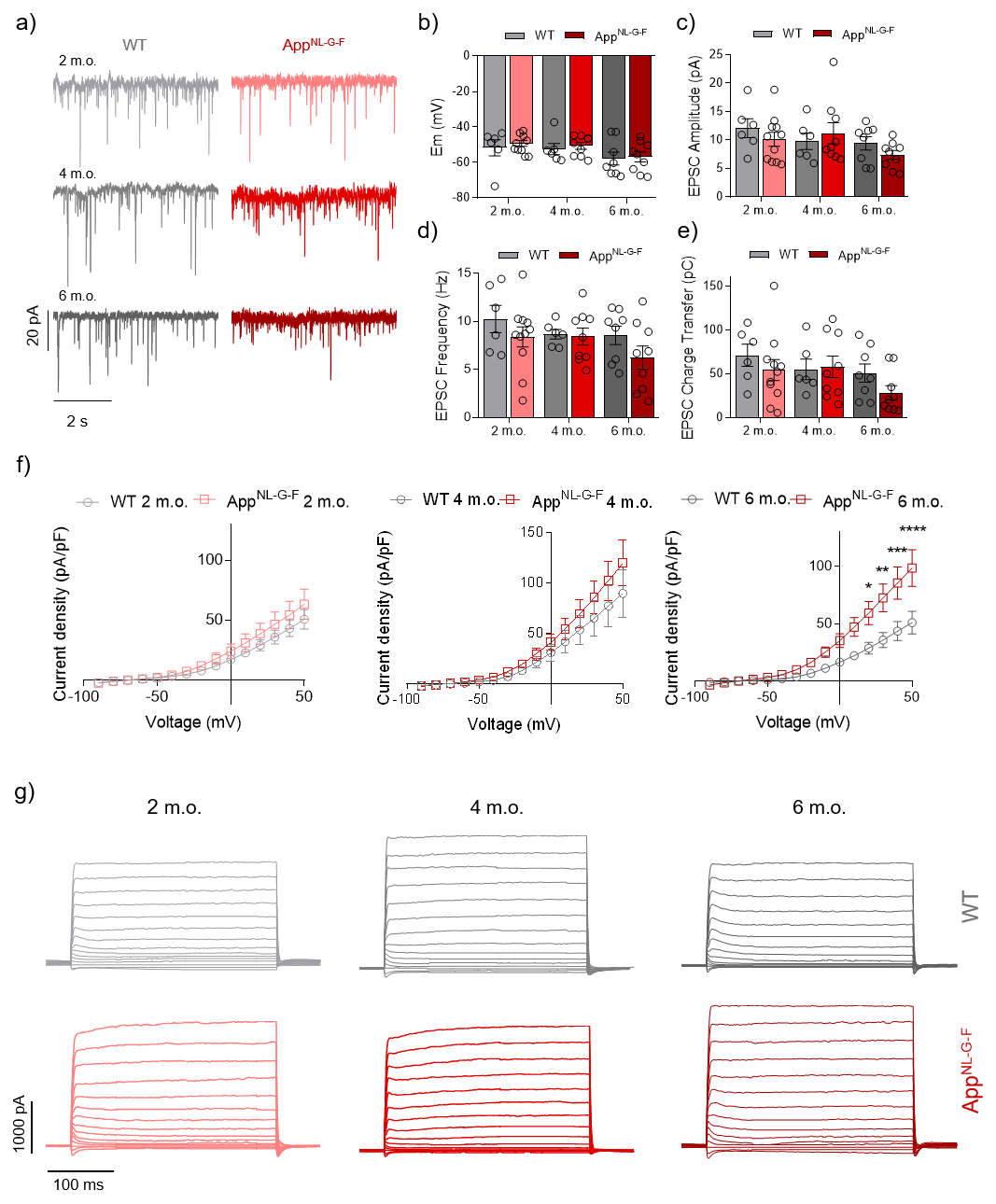

Supplement: Supplementary file 2 — Supplementary Figure 1. [file 41380_2021_1257_MOESM2_ESM.tif]

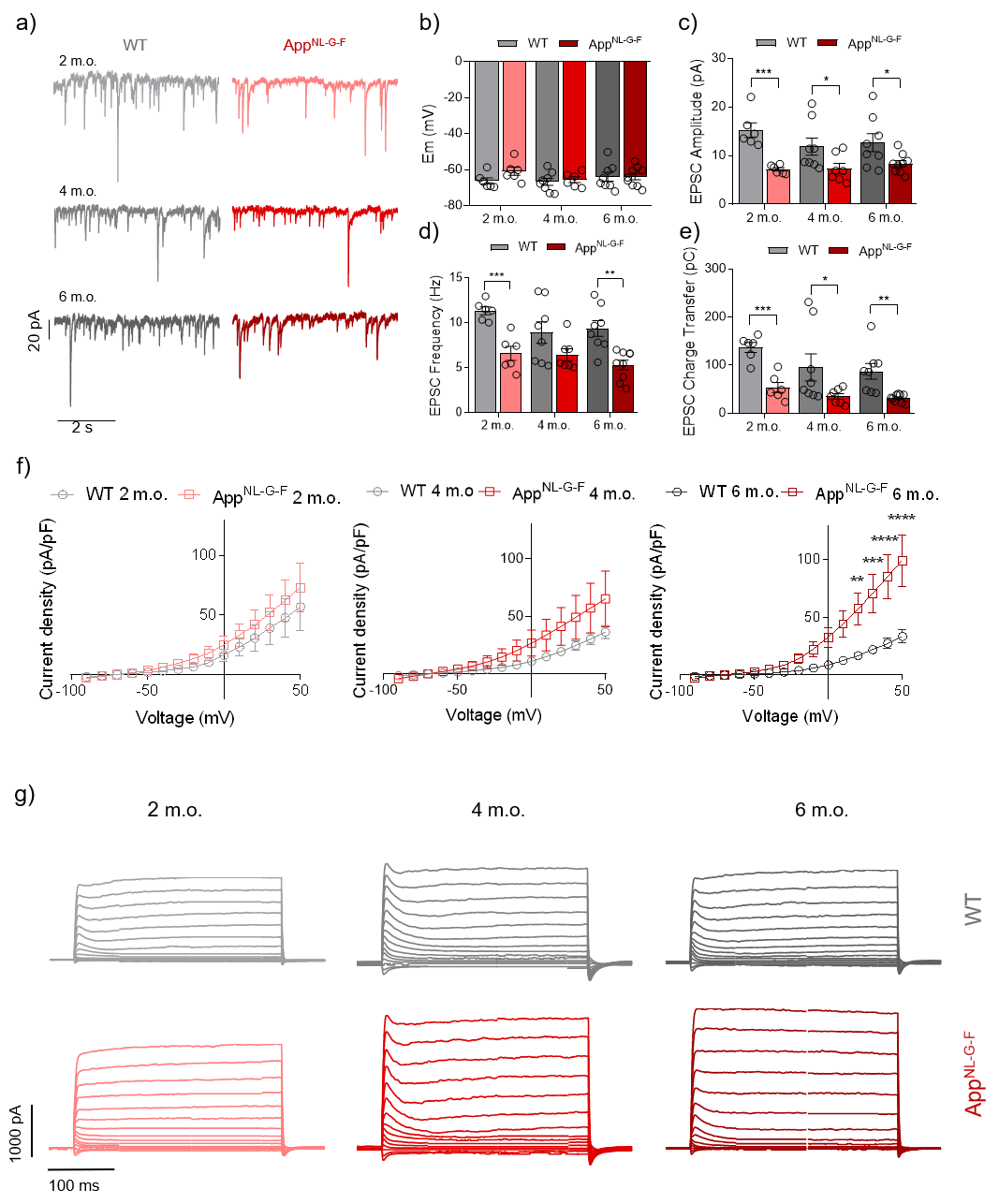

Supplement: Supplementary file 3 — Supplementary Figure 2. [file 41380_2021_1257_MOESM3_ESM.tif]

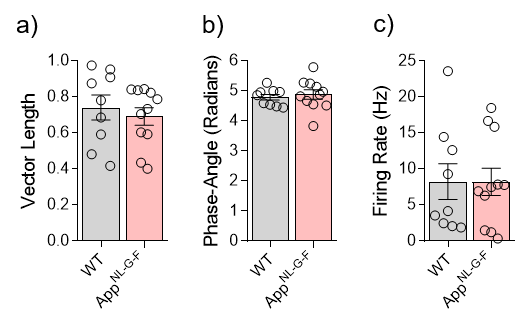

Supplement: Supplementary file 4 — Supplementary Figure 3. [file 41380_2021_1257_MOESM4_ESM.tif]

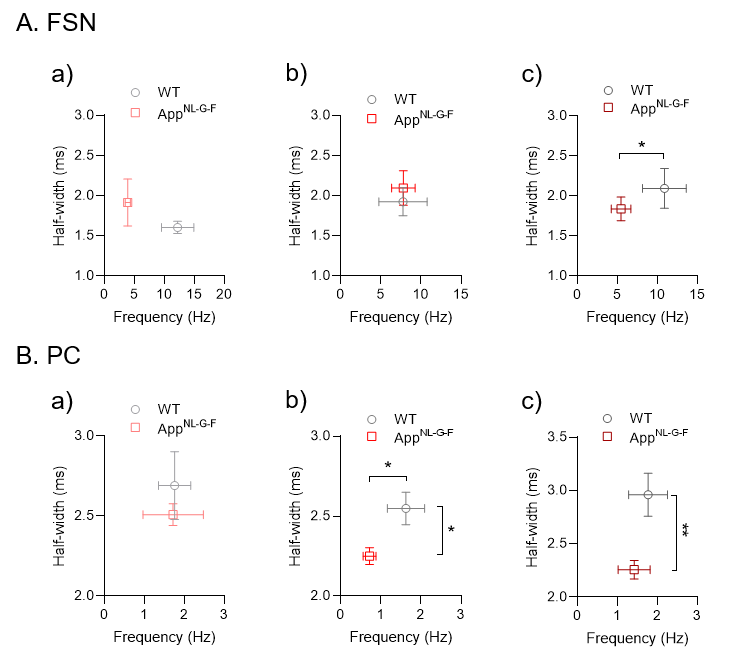

Supplement: Supplementary file 5 — Supplementary Figure 4. [file 41380_2021_1257_MOESM5_ESM.tif]

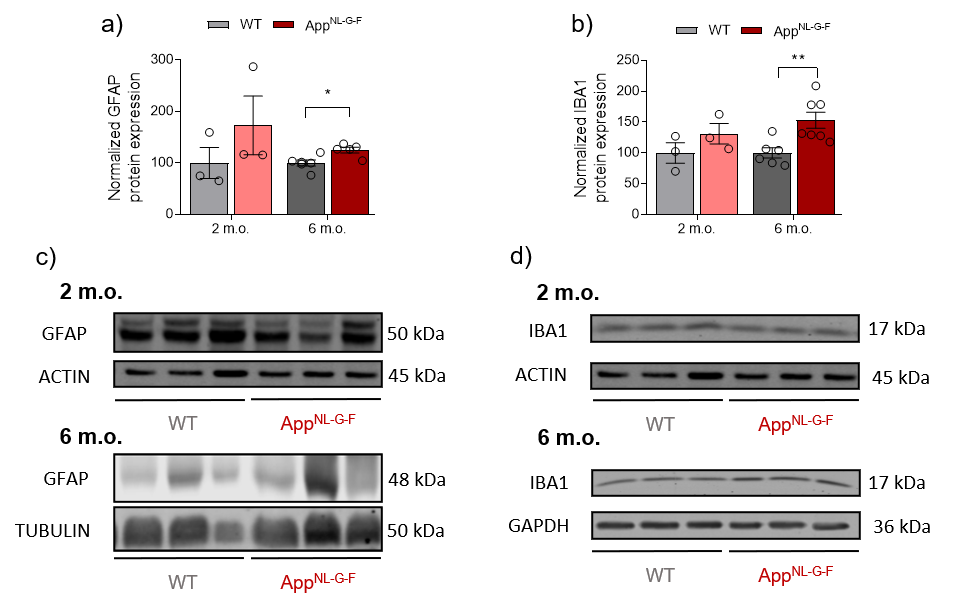

Supplement: Supplementary file 6 — Supplementary Figure 5. [file 41380_2021_1257_MOESM6_ESM.tif]
